# Assessment of Four Engineered PET Degrading Enzymes Considering Large-Scale Industrial Applications

**DOI:** 10.1021/acscatal.3c02922

**Published:** 2023-09-26

**Authors:** Grégory Arnal, Julien Anglade, Sabine Gavalda, Vincent Tournier, Nicolas Chabot, Uwe T. Bornscheuer, Gert Weber, Alain Marty

**Affiliations:** † Carbios, Parc Cataroux—Bâtiment B80, 8 Rue de la Grolière, 63100 Clermont-Ferrand, France; ‡ Toulouse Biotechnology Institute, TBI, Université de Toulouse, CNRS, INRAE, INSA, 135 Avenue de Rangueil, 31077 Toulouse Cedex 4, France; § Institute of Biochemistry, Biotechnology & Enzyme Catalysis, University of Greifswald, Felix-Hausdorff-Str. 4, 17487 Greifswald, Germany; ∥ Macromolecular Crystallography, Helmholtz-Zentrum Berlin für Materialien und Energie, Albert-Einstein-Straße 15, 12489 Berlin, Germany

**Keywords:** polyethylene terephthalate (PET), polyethylene terephthalate hydrolases, industrial enzymatic PET recycling, enzyme engineering, PET hydrolysis reaction conditions

## Abstract

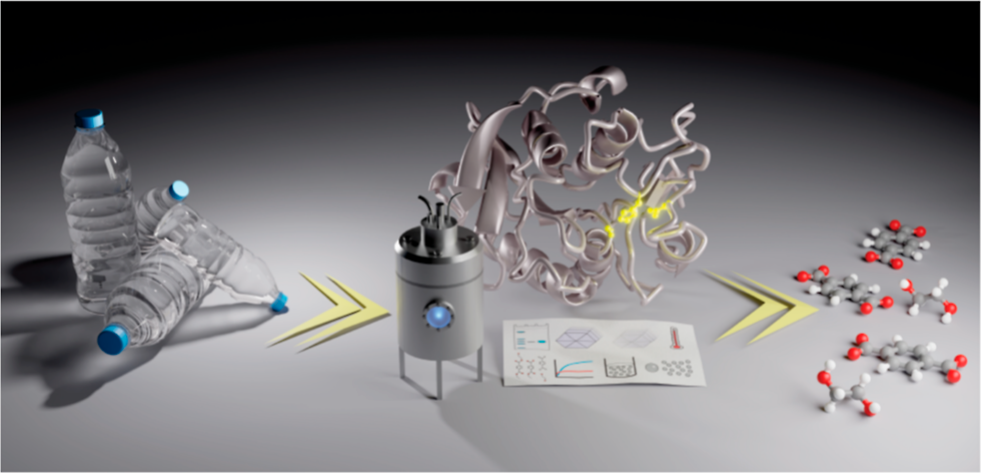

In recent years, enzymatic recycling of the widely used polyester polyethylene terephthalate (PET) has become a complementary solution to current thermomechanical recycling for colored, opaque, and mixed PET. A large set of promising hydrolases that depolymerize PET have been found and enhanced by worldwide initiatives using various methods of protein engineering. Despite the achievements made in these works, it remains difficult to compare enzymes’ performance and their applicability to large-scale reactions due to a lack of homogeneity between the experimental protocols used. Here, we pave the way for a standardized enzymatic PET hydrolysis protocol using reaction conditions relevant for larger scale hydrolysis and apply these parameters to four recently reported PET hydrolases (LCC^ICCG^, FAST-PETase, HotPETase, and PES-H1^L92F/Q94Y^). We show that FAST-PETase and HotPETase have intrinsic limitations that may not permit their application on larger reaction scales, mainly due to their relatively low depolymerization rates. With 80% PET depolymerization, PES-H1^L92F/Q94Y^ may be a suitable candidate for industrial reaction scales upon further rounds of enzyme evolution. LCC^ICCG^ outperforms the other enzymes, converting 98% of PET into the monomeric products terephthalic acid (TPA) and ethylene glycol (EG) in 24 h. In addition, we optimized the reaction conditions of LCC^ICCG^ toward economic viability, reducing the required amount of enzyme by a factor of 3 and the temperature of the reaction from 72 to 68 °C. We anticipate our findings to advance enzymatic PET hydrolysis toward a coherent assessment of the enzymes and materialize feasibility at larger reaction scales.

## Introduction

Despite the many benefits of synthetic polymers (plastics), their inadequate end-of-life management is a global threat to the environment, affecting ecosystems globally and posing a serious health warning.^1–3^ Polyethylene terephthalate (PET) is one of the most important polymers in terms of volume and accounts for 18% of the global plastic production.^4,5^ Current PET thermomechanical recycling strategies have significant drawbacks,^6^ such as limited waste sourcing (i.e., reliance on transparent bottles) and a decrease in their mechanical properties during the extrusion process. Consequently, more sustainable solutions that are in line with a circular economy are urgently needed. PET, composed of monomers linked by ester bonds, can be enzymatically hydrolyzed, yielding the products terephthalic acid (TPA) and ethylene glycol (EG), which are suitable for a resynthesis of the polymer after their purification. Nearly 20 years ago, the first hydrolase, a cutinase, was shown to specifically depolymerize PET.^7,8^ Since then, many other hydrolases have been isolated and enhanced through protein engineering.^4,9–11^ In 2020, leaf-branch compost cutinase (LCC^12^) was engineered into a quadruple variant called LCC^ICCG^ to meet industrial requirements.^13^ This study showed that monomers obtained through enzymatic hydrolysis under industry-relevant conditions could be purified and reused to obtain virgin PET, paving the way for the industrial deployment of enzyme-based PET depolymerization. Recently, FAST-PETase^14^ and HotPETase,^11^ two engineered variants of the poorly thermostable *Is*PETase^15^ from the bacterium *Ideonella sakaiensis*, were reported to show better PET hydrolyzing performances than LCC^ICCG^. Last, PES-H1^L92F/Q94Y^, a double variant of a metagenome-derived cutinase, was also shown to be a promising candidate for the deployment of enzyme-based PET recycling solutions.^16^ A direct comparison of the catalytic performances and potentials for larger scale applications remained very limited since the experimental parameters of all of these studies were widely different. Moreover, to adequately translate these enzymatic performances into a relevant large-scale industrial deployment, a list of key parameters should also be considered.^4,17,18^ Such key parameters are (i) PET crystallinity and associated pretreatment, (ii) surface of exchange and associated pretreatment, (iii) temperature of the enzyme-based PET depolymerization accounting for Arrhenius’ law, polymer’s glass transition temperature (*T*_g_), thermal induced crystallization, and enzyme thermostability, (iv) enzyme catalytic efficiency, to compete with PET crystallization kinetics, (v) PET concentration in the reactor, (vi) yield of the enzyme-based PET depolymerization, (vii) composition of the final products, and (viii) enzyme expressability (Table 1). The characteristics of PET used, its molar mass, its crystallinity, and the presence of comonomers such as isophthalic acid (IPA),^4^ as well as the shape and size of the degraded PET object (e.g., film or powder with a given particle size) are decisive factors. Many studies have demonstrated that PET hydrolases preferentially act on the amorphous regions of PET,^19–21^ and to the best of our knowledge, no PET hydrolases have been reported to act efficiently on highly crystalline forms of the polymer, typically found in consumer products.^4,22^ It thus appears crucial to perform a feedstock pretreatment to transform semicrystalline PET to its amorphous state in order to reach the high level of PET conversion (>90%) necessary to meet techno-economic goals^23^ and to meet process-based life cycle assessment of virgin PET production^24^ (Table 1). Another key parameter is the exchange surface between the solid plastic and the enzyme. The finer the particle size of the plastic powder, the faster the depolymerization kinetics will be.^25–27^ Such pretreatment appears mandatory for the industrial deployment of a PET recycling process in order to achieve high kinetics and yields,^28^ even if it has a negative impact on both capital and operational expenditures (CAPEX and OPEX)^23^ (Table 1). Enzyme thermostability is also a crucial parameter to attain high productivity, as, beyond the effect of the Arrhenius’ law, a high reaction temperature near the *T*_g_ increases the mobility of polymer chains^29–34^ (Table 1). However, two competing key events take place close to the *T*_g_: the kinetics of PET hydrolysis and the kinetics of PET recrystallization, the latter being counterproductive for efficient depolymerization. Thus, a thermostable enzyme must have sufficient catalytic efficiency to compete with the recrystallization rates (Table 1). Another important aspect of enzyme-based PET depolymerization on an industrial scale is that hydrolysis will not be performed in a dedicated buffered system but in water, mainly to simplify downstream processing but also to minimize OPEX. Evaluation of engineered enzymes’ performances should therefore be performed at low salt concentrations. Also, pH regulation is mandatory to ensure stable catalytic performance around enzymes’ pH optima (e.g., pH 7 to 9) and will be ensured by the addition of a base (e.g., NaOH) to neutralize the acidic products released during PET depolymerization (e.g., the diacid TPA and the monoacid MHET). Such base addition will lead to the formation of soluble disodium terephthalate, which will be recovered for further TPA purification. Maximization of the productivity per batch requires a high initial concentration of PET waste. This latter is dictated (taking into account base addition, transformation of PET solid polymer into two water-soluble products, and water addition for postreaction treatment) by the solubility of this terephthalate salt which is around 13% (w/w) between 25 and 70 °C.^35^ If the terephthalate salt solubility is exceeded, precipitated disodium terephthalate will be mixed with other insoluble products (remaining PET and other solid contaminants), rendering its recovery difficult. Additionally, considering the high price of PET waste and the cost of postreactional waste treatment, a minimum PET conversion of 90% (ideally 95%) must be reached to meet the expectations of an economically viable industrial recycling process^28^ (Table 1).

**Table 1:**
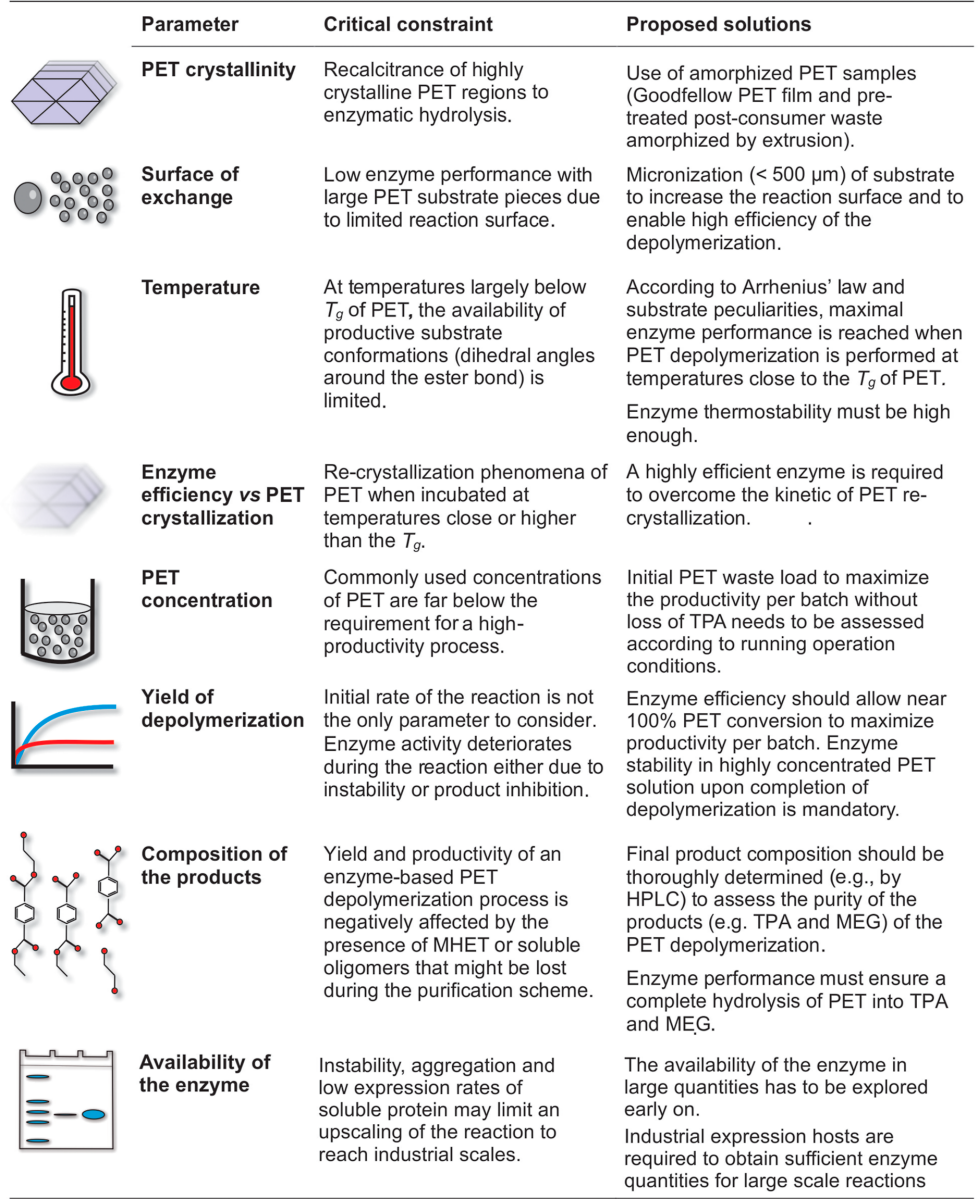
Critical Parameters to Consider for an Upscaling of the Enzyme-Based PET Depolymerization Reaction from the Perspective of an Industrial Deployment^a^

a Icons on the first column depict the parameter (second column) that is a critical constraint to be considered (third column) or its respective proposed solution (fourth column).

This high PET conversion must lead to the exclusive formation of TPA and EG while avoiding the accumulation of intermediate products (e.g., MHET) that would be lost during the purification scheme if a dedicated postdepolymerization treatment is not amended^14^ (Table 1). Finally, high enzyme expressability (e.g., >20 g L^–1^ of extracellular protein) is crucial when operating an industrial unit, mainly for OPEX considerations, but only a few industrial contractors can achieve an appreciable expression yield using dedicated industrial hosts (Table 1). This specific aspect of enzyme expressability could not be assessed in our study and remains difficult to predict.

Taking all these industrial key parameters into consideration, we evaluated the performances of four recently reported pioneering PET hydrolases, which are LCC^ICCG^,^13^ FAST-PETase,^14^ HotPETase,^11^ and PES-H1^L92F/Q94Y^.^16^ First, their respective PET depolymerization performances were evaluated using a standardized substrate (micronized amorphous Goodfellow film) at five different reaction temperatures matching the optimum conditions reported in the corresponding studies. Furthermore, larger scale setups using amorphized micronized postconsumer PET bottle flakes were implemented as a proof of principle for economic feasibility. We found that FAST-PETase and HotPETase performances were markedly deteriorated once evaluated in the industrial-process-relevant–experimental setup used here and that LCC^ICCG^ outperformed all other enzymes tested. We conclude that a standardization of experimental reaction parameters of enzymatic PET degradation, as provided in this article, is advisable to advance enzymatic PET hydrolysis toward industrial applications.

## Results

### Standardized Assay to Evaluate Performances of Four Engineered PET Degrading Enzymes

**Figure 1:**
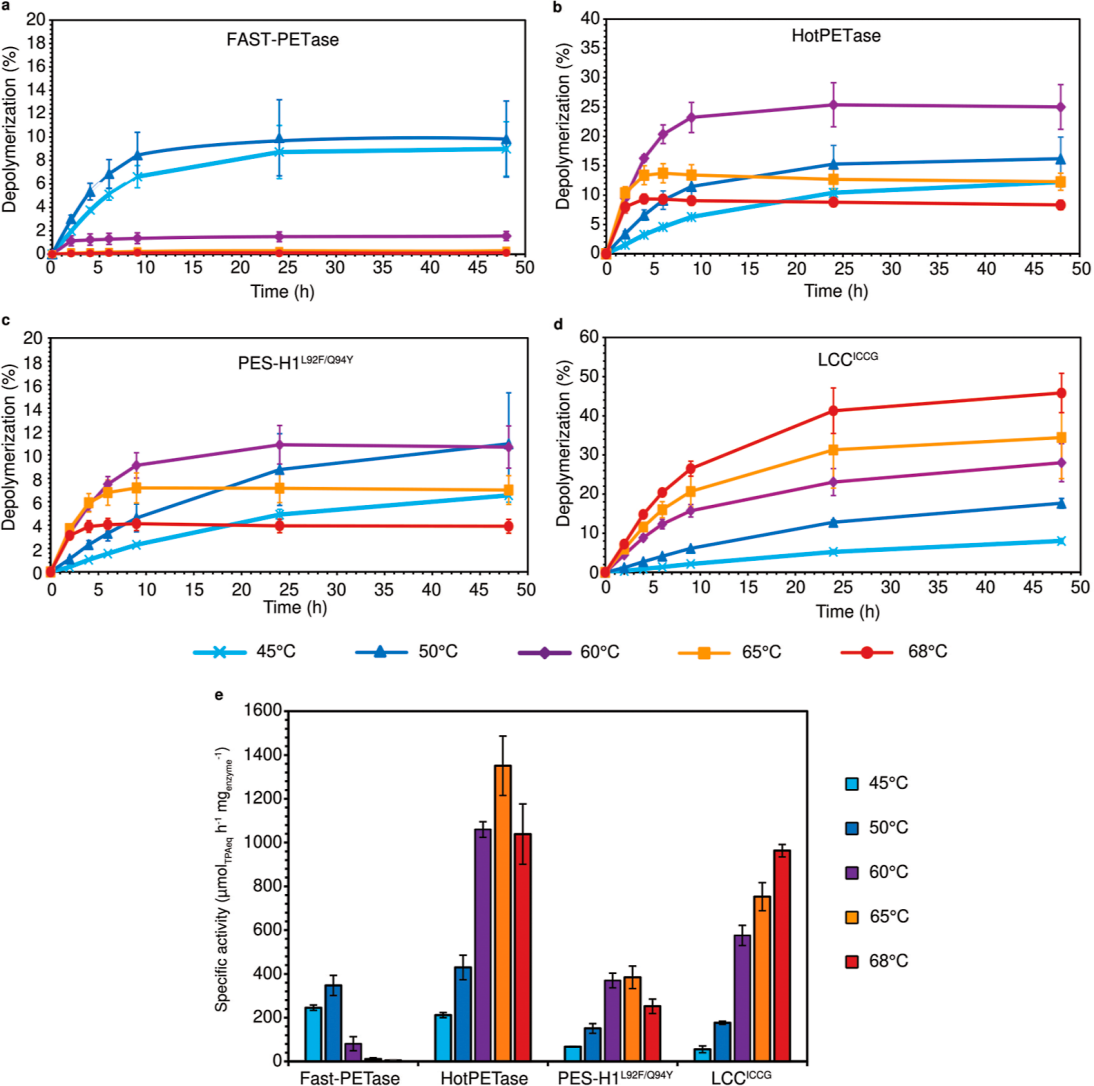
Performance assessments of four enzymes during Gf-PET depolymerization employing a unified and universally applicable assay format. Enzyme-based PET depolymerizations of a 2 g_PET_ L^–1^ solution (total volume of 50 mL) under nonsaturating concentration of enzyme (0.2 mg_enzyme_ g_PET_^–1^) performed at 45 °C (light blue, cross), 50 °C (blue, filled triangle), 60 °C (purple, filled diamond), 65 °C (orange, filled square) and 68 °C (red, filled circle) for (a) FAST-PETase (pH 8.0), (b) HotPETase (pH 9.2), (c) PES-H1^L92F/Q94Y^ (pH 8.0), and (d) LCC^ICCG^ (pH 8.0). (e) Specific activities of the four enzymes were assessed from the PET depolymerizations performed at different temperatures. Mean ± s.d. (*n* = 3).

Numerous strategies have successfully been developed to improve PET hydrolase performances in heterogeneous systems. Nevertheless, there is still a lack of a standardized approach to rationalize the kinetics of these interfacial enzymes, therefore hampering fundamental and comparative descriptions of PET-hydrolases.^36^ Amorphous Goodfellow film is a commercially available substrate suitable to compare enzyme performances. Exchange surface is a key parameter for the efficient hydrolysis of PET, and several PET hydrolases have shown very poor performances on amorphous PET film compared to amorphous PET powder.^22^ Consequently, cryo-ground Goodfellow films sieved under 500 μm (Gf-PET) appear as good candidates for further enzymatic reactions. Comparative performance assessments were performed using an initial PET concentration of 2 g_PET_ L^–1^ (e.g., 100 mg of Gf-PET in 50 mL) in a low ionic strength buffer but sufficient to keep a stable pH over a theoretically full PET conversion, where 10.4 mM of TPA would be released. To facilitate the evaluation of the four different biocatalysts studied here (LCC^ICCG^,^13^ FAST-PETase,^14^ HotPETase,^11^ and PES-H1^L92F/Q94Y^),^16^ the enzyme/substrate ratio (e.g., mg_enzyme_ g_PET_^–1^), the specific activity (e.g., μmol_TPAeq_ h^–1^ mg_enzyme_^–1^), as well as the final PET conversion obtained after a given time of enzyme treatment are provided as already pointed out in recent reviews.^37–39^ All biocatalysts were purified from *Escherichia coli* as the expression host (Figure S1) and subjected to differential scanning fluorimetry (DSF) for melting temperature assessments (Figure S2). The evaluated *T*_m_ matched the literature values (Table S1). FAST-PETase has the lowest melting temperature (63.3 °C), followed by PES-H1^L92F/Q94Y^ (77.6 °C), HotPETase (80.5 °C), and LCC^ICCG^ (91.7 °C). As mentioned above, it is very important to perform PET depolymerization at a high temperature, close to the *T*_g_ of PET which is near 70 °C^40–43^ in aqueous solution. Enzymes’ performances were evaluated at low (nonsaturating) enzyme concentration (0.2 mg_enzyme_ g_PET_^–1^) using 2 g_PET_ L^–1^ and at five temperatures to match the optimal temperature range reported in the literature for each enzyme (e.g., 45, 50, 60, 65, and 68 °C). The maximal temperature of 68 °C was chosen to avoid PET recrystallization, which impedes enzymatic PET hydrolysis for all known enzymes. In the light of the literature, performances of FAST-PETase, PES-H1^L92F/Q94Y^, and LCC^ICCG^ were evaluated using 0.1 M phosphate buffer at pH 8.0 while performances of HotPETase were monitored using 0.05 M glycine–OH buffer at pH 9.2, as reported in the earlier study.^11,13,14,16^ Kinetics of PET depolymerization were assessed using UV absorbance analysis, where all soluble products released during PET depolymerization (e.g., TPA, MHET, BHET, and longer soluble oligomers) can be accounted for. The results of these PET depolymerizations are shown in Figure 1a–d and the assessed enzymes’ specific activities (SA) are shown in Figure 1e.

FAST-PETase has a SA of 246 μmol_TPAeq_ h^–1^ mg_enzyme_^–1^ when PET depolymerization is performed at 45 °C and exhibited its highest SA at 50 °C, (348 μmol_TPAeq_ h^–1^ mg_enzyme_^–1^) (Figure 1e and Table S2) where the reaction reached a PET conversion of 10% of hydrolysis after 24 h before stopping the reaction (Figure 1a and Table S2). When PET depolymerization was performed at 60 °C and above, FAST-PETase suffered from its low thermostability and appeared to be destabilized very rapidly, with no significant activity detected. As expected for a more thermostable enzyme, the SA of HotPETase was improved from 211 μmol_TPAeq_ h^–1^ mg_enzyme_^–1^ at 45 °C up to 1351 μmol_TPAeq_ h^–1^ mg_enzyme_^–1^ at 65 °C (Figure 1e and Table S2). However, while 25% substrate conversion was achieved at 60 °C, only 12% was reached at 65 °C, reflecting HotPETases’ low stability at temperatures higher than 60 °C (Figure 1b and Table S2). Likewise, PES-H1^L92F/Q94Y^ showed its highest SA at 65 °C (481 μmol_TPAeq_ h^–1^ mg_enzyme_^–1^) (Figure 1e and Table S2), and depolymerization stopped rapidly at temperatures higher than 60 °C (Figure 1c). While the reaction stopped after 9 h to reach 7% PET depolymerization at 65 °C, it was able to run over 24 h at 60 °C to reach 11% PET conversion. Finally, LCC^ICCG^ showed its highest SA at 68 °C (963 μmol_TPAeq_ h^–1^ mg_enzyme_^–1^) (Figure 1e and Table S2), and its overall stability allowed the reaction to proceed over 48 h at 68 °C (Figure 1d), where PET conversion reached 46% (Table S2). From this first performance evaluation, we decided to use each enzyme under its best temperature condition (e.g., 50 °C for FAST-PETase, 60 °C for HotPETase and PES-H1^L92F/Q94Y^, and 68 °C for LCC^ICCG^) to reassess the enzyme performances under an upscaled PET depolymerization assay, including the new constraints previously described.

### Performances of the PET Hydrolases under Larger Scale Bioreactor Conditions

Gf-PET appears to be an appropriate uniform PET substrate accessible to everyone to perform a comparative evaluation of PET hydrolases through SA and stability studies. Nevertheless, it is of great interest to further corroborate these initial performances by conducting enzyme-based depolymerization on a larger scale (e.g., in a 0.5 L bioreactor) using a significant amount of postconsumer waste polymer (PcW-PET). Even though postconsumer bottle flakes are readily available globally from various suppliers, these PET samples can be produced by crushing a mix of water, soda, milk, or cosmetics bottles. Still, this crushed material is highly crystalline (typically 30–40% crystallinity), which makes it recalcitrant to enzymatic hydrolysis. A suitable amorphous material was obtained by rapidly cooling previously melted PET pellets in an extruder at 265 °C and cryogrinding them into sieved PET powder with a particle size of less than 500 μm.^5^

The investment expenditure (CAPEX) dedicated to the depolymerization section mainly relies on the productivity of the PET depolymerization, expressed in grams of products released per liter and per hour. This productivity is a function of three parameters: (i) the concentration of PET waste, (ii) the kinetics of the reaction, and (iii) the final conversion of PET and hence the yield of TPA and EG recovered, as underlined by a life cycle assessment study of enzymatic PET recycling.^24^ To maximize batch productivity, we aimed for a maximum PET loading in the reactor while ensuring that the disodium terephthalate generated would remain soluble. Considering the disodium terephthalate solubility (13% w/w),^35^ the addition of a 20% (w/w) NaOH solution for pH regulation and the transformation of PET solid polymer into two water-soluble products, the initial PET waste concentration can be set at 15.5% (w/w). However, within an industrial facility, an additional water volume must be considered to clean the reactor, to flush circuits from the reactor to the filter, and to wash the postreaction filtrate. Two loadings of PET were then tested: 16.5% (w/w) and 20% (w/w), considering a post reaction addition of water of 3.5 and 20%, respectively.

**Figure 2:**
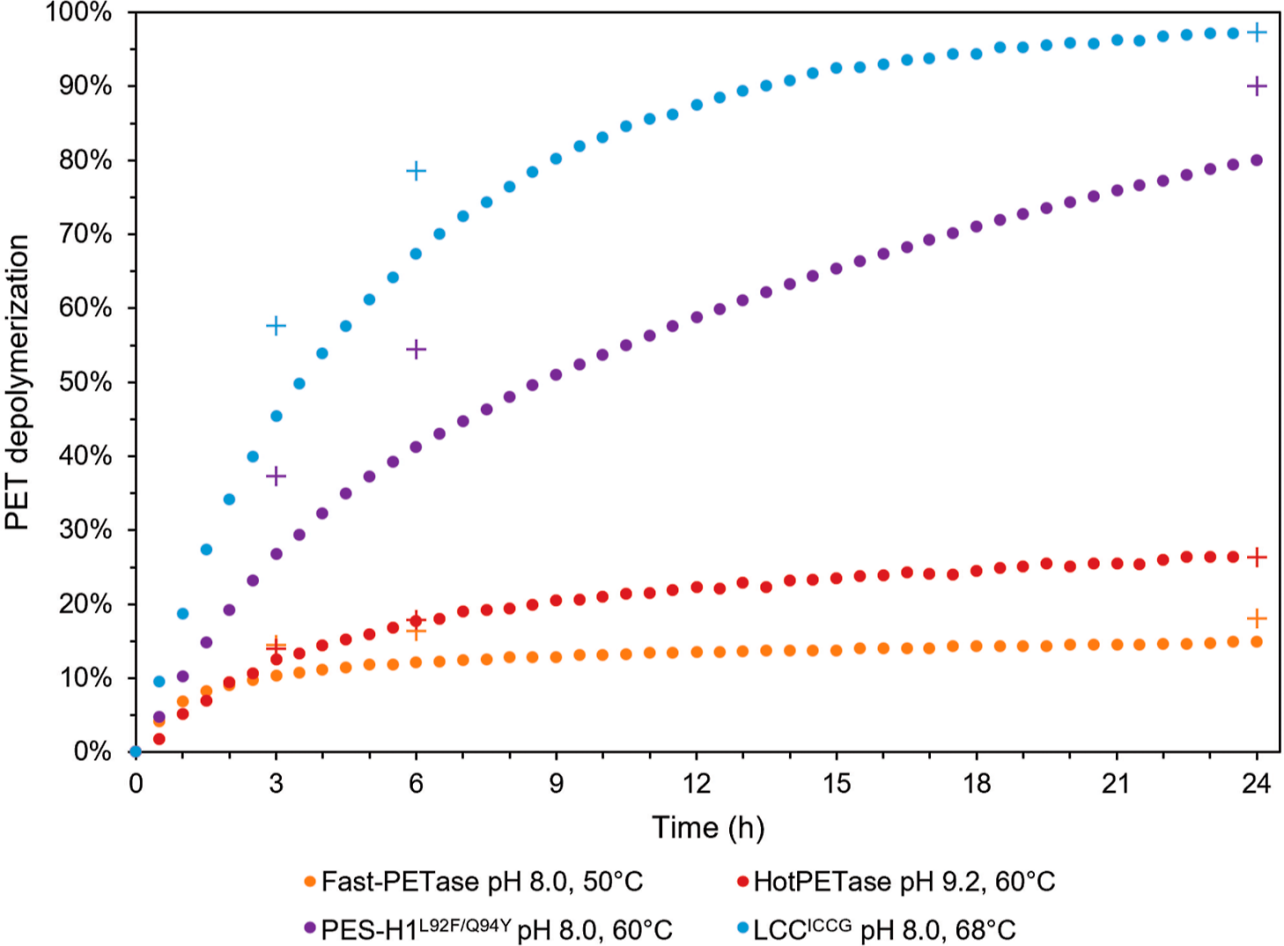
Comparison of 16.5% (w/w) PcW-PET depolymerizations performed by the four enzymes at bioreactor scale. Enzyme-based PET depolymerizations were performed using FAST-PETase at 50 °C, pH 8.0 (orange), HotPETase at 60 °C, pH 9.2 (red), PES-H1^L92F/Q94Y^ at 60 °C, pH 8.0 (purple), and LCC^ICCG^ at 68 °C, pH 8.0 (blue) of a 165 g_PET_ kg^–1^ solution with 1 mg_enzyme_ g_PET_^–1^. Dots represent the PET conversion in % measured by the NaOH consumption, considering an exclusive production of TPA and EG (2 mol of NaOH is consumed to titrate 1 mol of the diacid TPA). Crosses represent the percentage of PET conversion adjusted by considering the TPA/MHET ratio (1 mol of NaOH is consumed to titrate, 1 mol of the monoacid MHET).

Considering the enzymes’ performances described previously, 16.5% (w/w) PcW-PET depolymerizations were performed at 50 °C when using FAST-PETase, 60 °C for HotPETase and PES-H1^L92F/Q94Y^, and 68 °C for LCC^ICCG^ (Figure 2, Table S3). PET substrate conversion reached 15% after 24 h using FAST-PETase at 50 °C (Table S3), but the PET depolymerization rate slowed down considerably very early on (2 h after the beginning of the reaction), indicating insufficient stability of this enzyme even at 50 °C (Figure 2). Consequently, while Fast-PETase has a maximum productivity of 13.5 g_TPAeq_ L^–1^ h^–1^, the average productivity is only 0.9 g_TPAeq_ L^–1^ h^–1^ over 24 h (Table 2). Likewise, HotPETase was able to achieve 26% PET conversion in 24 h at 60 °C, but its activity deteriorated after 2 to 3 h of reaction time (Figure 2 and Table S3). A maximum productivity of 10.8 g_TPAeq_ L^–1^ h^–1^ can be estimated, but it decreases to an average productivity of 1.57 g_TPAeq_ L^–1^ h^–1^ over 24 h (Table 2). Despite all efforts devoted to the evolution of *Is*PETase, it appears that this enzyme still suffers from an intrinsic lack of (thermo)stability, and the catalytic properties of HotPETase, including product inhibition, are still not aligned with the requirements for its implementation in an enzyme-based PET depolymerization process on the industrial scale. In contrast, the 80% PET conversion obtained after 24 h (96% after 48 h) when using PES-H1^L92F/Q94Y^ at 60 °C (Figures 2, S3 and Table S3) emphasizes continuous enzyme performance when increasing the scale of the PET depolymerization assay. A maximum productivity of 15.5 g_TPAeq_ L^–1^ h^–1^ can be estimated as well as an average productivity of 4.75 g_TPAeq_ L^–1^ h^–1^ over 24 h (Table 2). Last, the highest efficiency in PET depolymerization was observed for LCC^ICCG^ converting 97% of the PET introduced into TPA and EG in 24 h (Figure 2 and Table S3) with an average productivity of 5.8 g_TPAeq_ L^–1^ h^–1^ and a maximum productivity of 28.6 g_TPAeq_ L^–1^ h^–1^ (Table 2).

**Table 2:** Productivities of the Four Different PET Hydrolases Using 16.5% (w/w) Post-Consumer Colored-Flake PET Waste Powder (PcW-PET) as Substrate

enzyme	maximum productivity [g_TPAeq_ L^–^^1^ h^–^^1^]	average productivity [g_TPAeq_ L^–^^1^ h^–^^1^]
FAST-PETase	13.5	0.9^a^
HotPETase	10.8	1.6^a^
PES-H1^L92F/Q94Y^	15.5	4.8^a^; 2.8^b^
LCC^ICCG^	28.6	5.8^a^

a At 24 h.

b At 48 h.

Notably, during the first hours of the reaction, MHET represents a significant amount of the degradation products (Figure 2 and Table S3). After around 3 h of reaction time using FAST-PETase, HotPETase, PES-H1^L92F/Q94Y^, and LCC^ICCG^, MHET accounts for 58, 21, 57, and 43% of the acidic products released (e.g., the diacid TPA and the monoacid MHET), respectively. Conversely, after 24 h, TPA becomes predominant and represents 64, 100, 78, and 100% of the soluble acidic products released, respectively. At 48 h, MHET represents only 1% of the total products released by PES-H1^L92F/Q94Y^. Using these values, and considering that 2 mol NaOH is used to neutralize 1 mol TPA while 1 mol NaOH is used to neutralize 1 mol MHET, 18.1, 26.4, 90.1, and 97.3% PET conversion were effectively reached after 24 h for FAST-PETase, HotPETase, PES-H1^L92F/Q94Y^, and LCC^ICCG^, respectively (Figures 2, S3 and Table S3), and 96.2% was reached after 48 h for PES-H1^L92F/Q94Y^. These values agree with PET conversion calculated from the residual dry weight assessment performed at the end of the reaction (Table S3) (18.6, 28.7, 98.6, and 98.1% when using FAST-PETase, HotPETase, PES-H1^L92F/Q94Y^, and LCC^ICCG^, respectively). Interestingly, when considering the whole reaction using LCC^ICCG^ at 68 °C, we noticed that 58 and 79% PET conversion can be achieved in only 3 and 6 h, respectively, illustrating that the last 18 h of the PET depolymerization is mostly dedicated to MHET hydrolysis. This MHET hydrolysis can be catalyzed by the enzyme, but it was also demonstrated to be due to its spontaneous hydrolysis.^44^ Such instability of the MHET might also be accelerated when PET depolymerization is performed at a higher pH, as it is the general rule for ester hydrolysis^45^ and could explain the lack of MHET accumulation observed during the PET depolymerization at pH 9.2 using HotPETase.

At 20% PcW-PET loading, the overall behavior is like the one observed at 16.5% for the four enzymes (Figures S4 and S5, Tables S4 and S5). With the exception of PES-H1^L92F/Q94Y^, the increase in productivity is linear between the two PET loadings. For PES-H1^L92F/Q94Y^, the productivity is slightly decreasing between 16.5 and 20% loading (from 4.8 to 4.4 g_TPAeq_ L^–1^ h^–1^ at 24 h; from 2.8 to 2.7 g_TPAeq_ L^–1^ h^–1^ at 48 h).

## Discussion and Conclusions

In the past few years, numerous publications dealing with the discovery and engineering of PET hydrolases have been released. To improve the activity and thermostability of such PETases, various concepts of protein engineering were implemented, such as rational design,^13^ directed evolution,^11^ or AI-assisted in silico protein design.^14^ The field has greatly benefited from these studies, since they have opened a plethora of new possible avenues for enzymatic PET recycling. Unfortunately, a thorough comparison of enzyme performance remains problematic since no international scientific consensus for the evaluation of PET hydrolase performance has emerged. The nature and properties of the substrates used can differ as well as reaction conditions, analytical methods, and more importantly readout parameters. In our study, we targeted over 90% (ideally 95%) PET depolymerization from the perspective of a viable large-scale biotechnological process development. We postulated that studies solely relying on enzyme specific activity as readouts were unable to assess the true applicability of the enzymes for industrial deployment. One of the reasons is very likely the number of new constraints to be considered on a larger scale. As a first step, we have therefore performed a standardized small-scale PET depolymerization study of four engineered PET hydrolases (e.g., FAST-PETase,^14^ HotPETase,^11^ PES-H1^L92F/Q94Y^^16^, and LCC^ICCG^^13^) to evaluate their optimal temperature condition. Such standardization comprised the use of cryo-ground amorphous commercial Goodfellow film with known particle size distribution, a fixed enzyme/substrate ratio (mg_enzyme_ g_PET_^–1^), and a fixed polymer concentration (g_PET_ L^–1^). Enzymes’ performances were evaluated at such small scale by determining standardized parameters such as final PET conversion/terephthalic acid formed after a given time and enzyme-specific activity values (μmol_TPAeq_ h^–1^ mg_enzyme_^–1^ or g_TPAeq_ h^–1^ mg_enzyme_^–1^) as recommended elsewhere.^37^ Then, a thorough comparison of the four PET hydrolases was performed in bioreactors using high concentrations of pretreated postconsumer PET waste material (165 and 200 g_PET_ kg^–1^) to mimic the conditions of an industrial process. Parameters such as the average and maximum productivity (expressed in g_TPAeq_ L^–1^ h^–1^), the PET conversion/terephthalic acid formed after a given time, and the purity of the products (e.g., TPA, MHET) were carefully assessed. We find that a typical weakness of the PET degrading enzymes is the deterioration of the activity over a long–time reaction that is in turn required to achieve 100% substrate conversion. FAST-PETase showed convincing performances in terms of specific activities at both reactor scales, but the kinetics of PET conversion in the reactor dramatically slowed down to reach less than 20% at 24 h. This observation appears to be in-line with the previous study where FAST-PETase enabled a 90% PET depolymerization from a 45 g_PET_ L^–1^ solution (e.g., amorphized PET bottle flakes) after 14 days at 50 °C but necessitating a daily replenishing of fresh enzyme solution (i.e., replacing the entire reaction solution) to compensate for the low stability of the enzyme.^14^ HotPETase, a more thermostable variant of the *Is*PETase, can achieve a higher conversion of 26% at 24 h. Likewise, Bell et al. have shown a substantial decrease in PET conversion after 5 h of reaction using HotPETase correlated to enzyme deactivation but not to product inhibition or PET.^11^ Comparatively, PES-H1^L92F/Q94Y^ and LCC^ICCG^ appeared more stable over time in reactor conditions, and near-complete PET conversions (∼98%) were attained within 48 and 24 h, respectively, at 16.5% PET loading (81 and 98% PET conversion at 20% PET loading, respectively). LCC^ICCG^ and PES-H1^L92F/Q94Y^ thus appear as good candidates for the deployment of an enzyme-based-PET-depolymerization process.

The behavior of PES-H1^L92F/Q94Y^ in the reactor is intriguing. Its *T*_m_ is lower than that of HotPETase (77.6 and 80.7, respectively) and in small reactors, at low PET concentration, and in nonsaturating enzyme conditions, the reaction stops rapidly at less than 12% conversion. This contradiction cannot be simply explained by low stability or inhibition by the products. Further investigations are necessary to solve this mystery.

Since the last study describing LCC^ICCG^ performance,^13^ reaction conditions have been largely improved in this work. While 95% PET conversion was previously obtained after 24 h using 3 mg_enzyme_ g_PET_^–1^ at 72 °C,^13^ 98% PET conversion was obtained here by introducing 1 mg_enzyme_ g_PET_^–1^ at 68 °C. Reducing the temperature of the reaction by 4 °C enabled the PET recrystallization kinetics to be reduced, which favors obtaining high conversion. The positive outcome was a reduction of the amount of enzyme by a factor of 3 while increasing the final PET conversion in the same time frame and consequently decreasing the cost of waste treatment. Such enzyme performance is meeting one of the bottlenecks described in a comprehensive LCA study of enzymatic recycling of PET, highlighting the need to achieve conversions greater than 90% at high PET concentrations to minimize postreaction waste.^24,28^ Finally, TPA and EG monomers are exclusively produced without the accumulation of MHET in the solution, simplifying the purification scheme of the products.

We believe that our study of PET hydrolases will contribute to finding a consensus on the methods used and key parameters to consider when performing a large-scale PET depolymerization. Such a consensus is necessary for the deployment of the first generation of enzymatic PET recycling processes at an industrial scale. We also believe that this field will further benefit from new technologies to emerge as well as new superior enzymes being able, for instance, to perform efficient degradation of semicrystalline PET to minimize the PET pretreatment steps as underlined in a previous study.^28^ Ultimately, new acid-tolerant PET hydrolases able to perform efficient PET depolymerization with no (or a minimal) need of soda for pH regulation would also be of great interest to further strengthen the concept of enzymatic PET hydrolysis in the frame of a circular economy.

## Materials and Methods

### PET Powder Preparation

Amorphous commercial PET was provided by Goodfellow Cambridge Ltd. (Huntingdon, UK, product number ES301445) and postconsumer colored-flake PET waste were provided by Sorepla Technologie SA, a recycling company (Neufchâteau, France). Amorphous commercial PET powder (Gf-PET) and postconsumer colored-flake PET waste powder (PcW-PET), with 98% PET content, were prepared as previously described.^13^ Gf-PET material has a *T*_g_ of 76.5 °C, a percentage of crystallinity of 7.7%, and is constituted of particles with sizes lower than 500 μm. PcW-PET material has a *T*_g_ of 78.4 °C, a percentage of crystallinity of 14.6%, and is constituted of particles with size lower than 500 μm (*D*_90_ < 400 μm and *D*_50_ between 200 and 250 μm).

### Gene Construction

The genes encoding PES-H1^L92F/Q94Y^,^16^ FAST-PETase,^14^ and HotPETase^11^ were synthesized with codon optimization for expression in *E. coli* cells (GeneCust, Boynes, France) into the pET-26b(+) (Novagen, San Diego, USA) vector between NdeI and XhoI restriction sites. The gene encoding for the leaf-branch compost cutinase (LCC) variant ICCG was cloned as described previously.^13^ A list of all nucleotide and amino acid sequences of the genes used in the study is provided in the Supporting Information.

### Preparative Protein Production

Genes were expressed in *E. coli* BL21 (DE3) competent cells (New England Biolabs, Ipswich, USA) by cultivation in ZYM auto-inducible medium^46^ for 23 h at 21 °C. The *E. coli* cells were harvested by centrifugation (6000*g*, 10 min, 10 °C) and suspended in lysis buffer (20 mM Tris–HCl, pH 8.0, 300 mM NaCl). Cells were disrupted by sonication on ice, and the lysate was clarified by centrifugation (10,000*g*, 30 min, 10 °C). The soluble fraction was applied to TALON metal affinity resin (Clontech, CA). After unbound proteins were washed with the lysis buffer supplemented by 10 mM imidazole, bound proteins were eluted with elution buffer (20 mM Tris–HCl, pH 8.0, 300 mM NaCl, 250 mM imidazole). The buffer was finally exchanged for storage buffer (100 mM potassium phosphate, pH 8.0 for LCC^ICCG^, FAST-PETase, and PES-H1^L92F/Q94Y^ or 50 mM glycine–OH buffer pH 9.2 for HotPETase) using Hiprep 26/10 desalting column (GE healthcare, Chicago, IL). Purified protein concentration was determined based on the calculated molar extinction coefficient at 280 nm. Protein purity was evaluated by SDS–PAGE analysis.

### Analytical Method for Melting Temperature Assessment

DSF was used to assess the thermostability of LCC^ICCG^, FAST-PETase, HotPETase, and PES-H1^L92F/Q94Y^ by determining their melting temperature (*T*_m_). Protein samples were prepared at a concentration of 6.25 μM and stored in buffer consisting of potassium phosphate (pH 8.0, 100 mM) for LCC^ICCG^, FAST-PETase, and PES-H1^L92F/Q94Y^, or in glycine–OH buffer 50 mM, pH 9.2 for HotPETase. The SYPRO Orange dye 5000× stock solution in DMSO was first diluted to 250× in water. Protein samples were loaded onto a white clear 96-well PCR plate (Lifescience Bio-Rad, France, catalog no. HSP9601) with each well containing a final volume of 25 μL. The final concentration of protein and SYPRO Orange dye in each well was 6 μM and 10×, respectively. Loaded volumes per well were as follows: 24 μL of the 6.25 μM protein solution and 1 μL of the 250× SYPRO Orange diluted solution. The PCR plates were then sealed with optical-quality sealing tape and spun at 2000 rpm for 1 min at room temperature. DSF experiments were then carried out using a Bio-Rad CFX96 real-time PCR system set on the FRET channel to use the 450/490 excitation and 560/580 emission filters. The samples were heated from 25 to 100 °C at the rate of 0.3 °C s^–1^. A single fluorescence measurement was taken every 0.03 s. The *T*_m_ was determined from the peak of the first derivatives of the melting curve using the Bio-Rad CFX Manager software. *T*_m_ values correspond to the average of three measurements.

### PET Depolymerization Assay Using Amorphous Goodfellow Film as Substrate

A 49 mL aliquot of potassium phosphate buffer (pH 8.0, 100 mM) or glycine–OH buffer (pH 9.2, 50 mM) was combined with 100 mg of Gf-PET in a 100 mL glass bottle and incubated at 45, 50, 60, 65, or 68 °C in a stirring dry bath 15–100 (2mag AG, Munich, Germany) under agitation at 200 rpm until the solution reached the desired temperature. The depolymerization was initiated by adding 1 mL of a 0.02 mg_enzyme_ mL^–1^ (0.7 μM of enzyme) solution of purified protein (final concentration of 0.2 mg_enzyme_ g_PET_^–1^) in 100 mM potassium phosphate buffer, pH 8.0 for LCC^ICCG^, FAST-PETase, and PES-H1^L92F/Q94Y^, or in glycine–OH buffer 50 mM, pH 9.2 for HotPETase. Samples were harvested at 2, 4, 6, 9, 24, 48, 72, and 96 h of reaction time and analyzed by ultraviolet light (UV) absorbance measurements at 242 nm for the determination of PET depolymerization kinetics (see below). Reactions were performed in triplicate.

### PET Depolymerization Assay in a Bioreactor

PET depolymerization reactions at 20% PET (w/w) were performed using 49 mg of purified protein (1.7 μmol) prepared in 195 mL of potassium phosphate buffer (pH 8.0, 100 mM) or glycine–OH buffer (pH 9.2, 50 mM) that was combined with 50 g of PcW-PET (98% purity). PET depolymerization reactions at 16.5% PET (w/w) were performed using 40.43 mg of purified protein (1.4 μmol) prepared in 203.75 mL of potassium phosphate buffer (pH 8.0, 100 mM) or glycine–OH buffer (pH 9.2, 50 mM) that were combined with 41.25 g of PcW-PET (98% purity). Reactions were performed in a 500 mL Benchtop F1 0.5 MB Bioreactor (AD Biotec, France). Temperature regulation was performed in the water-jacketed bioreactor, and a double Rushton impeller was used to maintain constant agitation at 800 rpm. The pH value was regulated to pH 8.0 or 9.2 by the addition of a 20% NaOH (w/w) solution using the ROSITA 2.0 software (AD Biotec, France). The kinetics of the PET depolymerization was followed based on NaOH consumption, considering the exclusive production of TPA and EG. Terephthalic acid has two carboxylic acid groups; therefore, 1 mol of NaOH titrates 0.5 mol of terephthalic acid. Thus, the conversion of PET to terephthalic acid can be easily calculated from the amount of NaOH consumed. In addition, samples were harvested at different time points and analyzed by UHPLC (see below) to adjust PET conversion overtime by considering the TPA/MHET ratio (1 mol of NaOH is consumed to titrate 1 mol of the monoacid MHET). The final yield of the PET depolymerization assay was determined either by NaOH consumption or by dry weight determination of residual PET. To determine dry weight of residual PET, the entire reaction solution, including solid particles, was filtered through a 12 to 15 μm grade 11 ashless paper filter (Dutscher SAS, Brumath, France) and dried. Maximum productivities in g_TPAeq_ L^–1^ h^–1^ were estimated from a linear time frame of the NaOH consumption kinetics generated using FAST-PETase, HotPETase, PES-H1^L92F/Q94Y^, or LCC^ICCG^. Average productivities in g_TPAeq_ L^–1^ h^–1^ were estimated after 24 h of reaction. An additional average productivity in g_TPAeq_ L^–1^ h^–1^ was specifically estimated after 48 h of reaction when using PES-H1^L92F/Q94Y^.

### Quantification of Soluble Products Using Ultraviolet Light Absorbance

Kinetics of Gf-PET enzymatic depolymerization were followed by UV light absorbance using a method adapted from Zhong-Johnson et al.^47^ Briefly, the absorbance of the reaction mixtures in the ultraviolet region of the light spectrum (at 242 nm) indicates the release of soluble TPA or its esters (MHET, BHET, and others) from the insoluble PET substrate. Standard curves of TPA, MHET, and BHET were performed at 242 nm using an Eon Microplate Spectrophotometer (BioTek, USA). An average coefficient of 16,400 M^–1^ cm^–1^ corresponding to a combination of these products was used. Samplings performed at different times (typically at 2, 4, 6, 9, 24, 48, 72, and 96 h) during the hydrolysis of Gf-PET were analyzed by absorbance reading at 242 nm. If necessary, samples were diluted in potassium phosphate buffer (pH 8.0, 100 mM). The absorbance value is used to calculate the overall sum of soluble PET hydrolysis products according to the Lambert–Beer law. The SA of PET hydrolysis in μmol_TPAeq_ h^–1^ mg_enzyme_^–1^ was determined in the linear part (typically between 0 and 4 h reaction time) of the hydrolysis curve of the reaction. Alternatively, when enzymes suffered from poor thermostability, specific activity was determined between 0 and 2 h of reaction time. The term TPA_eq_ corresponds to the sum of soluble products released from the hydrolysis the PET polymer (e.g., TPA, MHET, BHET, and longer soluble oligomers).

### Analytical Method for TPA, MHET, and BHET Detection by UHPLC

The concentrations of TPA, MHET, and BHET were monitored by UHPLC. When required, samples were diluted in potassium phosphate buffer (pH 8.0, 100 mM). Then, 150 μL of methanol and 6.5 μL of HCl 6 N were added to 150 μL of a (diluted) sample. After homogenization and filtering through a 0.45 μm syringe filter, 20 μL of the sample was injected into a UHPLC column. The chromatography system used was a Vanquish UHPLC system (Thermo Fisher Scientific, Waltham, MA) equipped with a pump module, an autosampler, a column oven thermostated at 25 °C, and a UV detector at 240 nm. TPA, MHET, and BHET were separated using a gradient of methanol (30 to 90%) in 1 mM H_2_S0_4_ at 1 mL min^–1^ through a Discovery HS C18 HPLC column (150 mm × 4.6 mm, 5 μm) equipped with a precolumn (Supelco, Bellefonte, PA). TPA, MHET, and BHET were quantified according to standard curves, prepared from commercial TPA and BHET (Sigma-Aldrich, St. Louis, MO) and in-house synthesized MHET,^13^ under the same conditions as for the samples.

## Supporting Information Available

SDS-PAGE analysis of 1- LCC^ICCG^, 2- FAST-PETase, 3- HotPETase, 4- PES-H1^L92F/Q94Y^; DSF thermal denaturation curves for FAST-PETase, PES-H1^L92F/Q94Y^, and LCC^ICCG^; 16.5% (w/w) PcW-PET depolymerization performed in a reactor using PES-H1^L92F/Q94Y^ over 48 h of hydrolysis; comparison of 20% (w/w) PcW-PET depolymerizations performed by the four enzymes at bioreactor scale; 20% (w/w) PcW-PET depolymerization performed in a reactor using PES-H1^L92F/Q94Y^ over 48 h of hydrolysis; T_*m*_ assessments of FAST-PETase, HotPETase, PES-H1^L92F/Q94Y^, and LCC^ICCG^; performances measured at different temperatures during the hydrolysis of Gf-PET for FAST-PETase, HotPETase, PES-H1^L92F/Q94Y^, and LCC^ICCG^; performances of FAST-PETase at pH 8.0, 50 °C, HotPETase at pH 9.2, 60 °C, PES-H1^L92F/Q94Y^ at pH 8.0, 60 °C, and LCC^ICCG^ at pH 8.0, 68 °C during the hydrolysis of 16.5% (w/w) PcW-PET in reactors; performances of FAST-PETase at pH 8.0, 50 °C, HotPETase at pH 9.2, 60 °C, PES-H1^L92F/Q94Y^ at pH 8.0, 60 °C, and LCC^ICCG^ at pH 8.0, 68 °C during the hydrolysis of 20% (w/w) PcW-PET in reactors; and productivities of the four different PET hydrolases using 20% (w/w) post-consumer colored-flake PET waste powder as substrate (PDF)

## Abbreviations

PET: polyethylene terephthalate
MHET: monohydroxyethylene glycol terephthalate
BHET: bis-hydroxyethylene glycol terephthalate
TPA: terephthalic acid
EG: ethylene glycol
IPA: isophthalic acid
LCC: leaf-branch compost cutinase

## References

[ref1] MacLeod M.; Arp H. P. H.; Tekman M. B.; Jahnke A. The Global Threat from Plastic Pollution. Science 2021, 373 (6550), 61–65. 10.1126/science.abg5433. 34210878

[ref2] Allen S.; Allen D.; Karbalaei S.; Maselli V.; Walker T. R. Micro(Nano)Plastics Sources, Fate, and Effects: What We Know after Ten Years of Research. J. Hazard. Mater. Adv. 2022, 6, 100057 10.1016/j.hazadv.2022.100057.

[ref3] Organisation for Economic Co-operation and Development. Global Plastics Outlook; OECD Publishing, 2022. 10.1787/de747aef-en.

[ref4] Tournier V.; Duquesne S.; Guillamot F.; Cramail H.; Taton D.; Marty A.; André I. Enzymes’ Power for Plastics Degradation. Chem. Rev. 2023, 123 (9), 5612–5701. 10.1021/acs.chemrev.2c00644. 36916764

[ref5] S&P Global. Chemical Economics Handbook—PET Polymer, 2021. https://www.spglobal.com/commodityinsights/en/ci/products/pet-polymer-chemical-economics-handbook.html.

[ref6] Ragaert K.; Delva L.; Van Geem K. Mechanical and Chemical Recycling of Solid Plastic Waste. Waste Manag. 2017, 69, 24–58. 10.1016/j.wasman.2017.07.044. 28823699

[ref7] Müller R. J.; Schrader H.; Profe J.; Dresler K.; Deckwer W. D. Enzymatic Degradation of Poly(Ethylene Terephthalate): Rapid Hydrolyse Using a Hydrolase from T.Fusca. Macromol. Rapid Commun. 2005, 26 (17), 1400–1405. 10.1002/marc.200500410.

[ref8] Alisch M.; Feuerhack A.; Müller H.; Mensak B.; Andreaus J.; Zimmermann W. Biocatalytic Modification of Polyethylene Terephthalate Fibres by Esterases from Actinomycete Isolates. Biocatal. Biotransform. 2004, 22 (5–6), 347–351. 10.1080/10242420400025877.

[ref9] Cui Y.; Chen Y.; Liu X.; Dong S.; Tian Y.; Qiao Y.; Mitra R.; Han J.; Li C.; Han X.; Liu W.; Chen Q.; Wei W.; Wang X.; Du W.; Tang S.; Xiang H.; Liu H.; Liang Y.; Houk K. N.; Wu B. Computational Redesign of a PETase for Plastic Biodegradation under Ambient Condition by the GRAPE Strategy. ACS Catal. 2021, 11 (3), 1340–1350. 10.1021/acscatal.0c05126.

[ref10] Rosenfeld L.; Heyne M.; Shifman J. M.; Papo N. Protein Engineering by Combined Computational and in Vitro Evolution Approaches. Trends Biochem. Sci. 2016, 41, 421–433. 10.1016/j.tibs.2016.03.002. 27061494

[ref11] Bell E. L.; Smithson R.; Kilbride S.; Foster J.; Hardy F. J.; Ramachandran S.; Tedstone A. A.; Haigh S. J.; Garforth A. A.; Day P. J. R.; Levy C.; Shaver M. P.; Green A. P. Directed Evolution of an Efficient and Thermostable PET Depolymerase. Nat. Catal. 2022, 5 (8), 673–681. 10.1038/s41929-022-00821-3.

[ref12] Sulaiman S.; Yamato S.; Kanaya E.; Kim J.-J.; Koga Y.; Takano K.; Kanaya S. Isolation of a Novel Cutinase Homolog with Polyethylene Terephthalate-Degrading Activity from Leaf-Branch Compost by Using a Metagenomic Approach. Appl. Environ. Microbiol. 2012, 78 (5), 1556–1562. 10.1128/AEM.06725-11. 22194294 PMC3294458

[ref13] Tournier V.; Topham C. M.; Gilles A.; David B.; Folgoas C.; Moya-Leclair E.; Kamionka E.; Desrousseaux M. L.; Texier H.; Gavalda S.; Cot M.; Guémard E.; Dalibey M.; Nomme J.; Cioci G.; Barbe S.; Chateau M.; André I.; Duquesne S.; Marty A. An Engineered PET Depolymerase to Break down and Recycle Plastic Bottles. Nature 2020, 580 (7802), 216–219. 10.1038/s41586-020-2149-4. 32269349

[ref14] Lu H.; Diaz D. J.; Czarnecki N. J.; Zhu C.; Kim W.; Shroff R.; Acosta D. J.; Alexander B. R.; Cole H. O.; Zhang Y.; Lynd N. A.; Ellington A. D.; Alper H. S. Machine Learning-Aided Engineering of Hydrolases for PET Depolymerization. Nature 2022, 604 (7907), 662–667. 10.1038/s41586-022-04599-z. 35478237

[ref15] Han X.; Liu W.; Huang J.-W.; Ma J.; Zheng Y.; Ko T.-P.; Xu L.; Cheng Y.-S.; Chen C.-C.; Guo R.-T. Structural Insight into Catalytic Mechanism of PET Hydrolase. Nat. Commun. 2017, 8 (1), 2106 10.1038/s41467-017-02255-z. 29235460 PMC5727383

[ref16] Pfaff L.; Gao J.; Li Z.; Jäckering A.; Weber G.; Mican J.; Chen Y.; Dong W.; Han X.; Feiler C. G.; Ao Y. F.; Badenhorst C. P. S.; Bednar D.; Palm G. J.; Lammers M.; Damborsky J.; Strodel B.; Liu W.; Bornscheuer U. T.; Wei R. Multiple Substrate Binding Mode-Guided Engineering of a Thermophilic PET Hydrolase. ACS Catal. 2022, 12 (15), 9790–9800. 10.1021/acscatal.2c02275. 35966606 PMC9361285

[ref17] Castro-Rodríguez J. A.; Rodríguez-Sotres R.; Farrés A. Determinants for an Efficient Enzymatic Catalysis in Poly(Ethylene Terephthalate) Degradation. Catalysts 2023, 13, 591 10.3390/catal13030591.

[ref18] Kushwaha A.; Goswami L.; Singhvi M.; Kim B. S. Biodegradation of Poly(Ethylene Terephthalate): Mechanistic Insights, Advances, and Future Innovative Strategies. Chem. Eng. J. 2023, 457, 141230 10.1016/j.cej.2022.141230.

[ref19] Marten E.; Müller R. J.; Deckwer W. D. Studies on the Enzymatic Hydrolysis of Polyesters I. Low Molecular Mass Model Esters and Aliphatic Polyesters. Polym. Degrad. Stab. 2003, 80 (3), 485–501. 10.1016/S0141-3910(03)00032-6.

[ref20] Wei R.; Breite D.; Song C.; Gräsing D.; Ploss T.; Hille P.; Schwerdtfeger R.; Matysik J.; Schulze A.; Zimmermann W. Biocatalytic Degradation Efficiency of Postconsumer Polyethylene Terephthalate Packaging Determined by Their Polymer Microstructures. Adv. Sci. 2019, 6 (14), 1900491 10.1002/advs.201900491. PMC6662049 31380212

[ref21] Kawai F.; Kawabata T.; Oda M. Current Knowledge on Enzymatic PET Degradation and Its Possible Application to Waste Stream Management and Other Fields. Appl. Microbiol. Biotechnol. 2019, 103, 4253–4268. 10.1007/s00253-019-09717-y. 30957199 PMC6505623

[ref22] Erickson E.; Gado J. E.; Avilán L.; Bratti F.; Brizendine R. K.; Cox P. A.; Gill R.; Graham R.; Kim D. J.; König G.; Michener W. E.; Poudel S.; Ramirez K. J.; Shakespeare T. J.; Zahn M.; Boyd E. S.; Payne C. M.; DuBois J. L.; Pickford A. R.; Beckham G. T.; McGeehan J. E. Sourcing Thermotolerant Poly(Ethylene Terephthalate) Hydrolase Scaffolds from Natural Diversity. Nat. Commun. 2022, 13 (1), 7850 10.1038/s41467-022-35237-x. 36543766 PMC9772341

[ref23] Singh A.; Rorrer N. A.; Nicholson S. R.; Erickson E.; DesVeaux J. S.; Avelino A. F. T.; Lamers P.; Bhatt A.; Zhang Y.; Avery G.; Tao L.; Pickford A. R.; Carpenter A. C.; McGeehan J. E.; Beckham G. T. Techno-Economic, Life-Cycle, and Socioeconomic Impact Analysis of Enzymatic Recycling of Poly(Ethylene Terephthalate). Joule 2021, 5 (9), 2479–2503. 10.1016/j.joule.2021.06.015.

[ref24] Uekert T.; DesVeaux J. S.; Singh A.; Nicholson S. R.; Lamers P.; Ghosh T.; McGeehan J. E.; Carpenter A. C.; Beckham G. T. Life Cycle Assessment of Enzymatic Poly(Ethylene Terephthalate) Recycling. Green Chem. 2022, 24 (17), 6531–6543. 10.1039/D2GC02162E.

[ref25] Castro A. M. D.; Carniel A.; Stahelin D.; Chinelatto Junior L. S.; Honorato H. d. A.; de Menezes S. M. C. High-Fold Improvement of Assorted Post-Consumer Poly(Ethylene Terephthalate) (PET) Packages Hydrolysis Using Humicola Insolens Cutinase as a Single Biocatalyst. Process Biochem. 2019, 81, 85–91. 10.1016/j.procbio.2019.03.006.

[ref26] Brizendine R. K.; Erickson E.; Haugen S. J.; Ramirez K. J.; Miscall J.; Salvachúa D.; Pickford A. R.; Sobkowicz M. J.; Mcgeehan J. E.; Beckham G. T. Particle Size Reduction of Poly(Ethylene Terephthalate) Increases the Rate of Enzymatic Depolymerization but Does Not Increase the Overall Conversion Extent. ACS Sustain. Chem. Eng. 2022, 10 (28), 9131–9140. 10.1021/acssuschemeng.2c01961.

[ref27] Gamerith C.; Zartl B.; Pellis A.; Guillamot F.; Marty A.; Acero E. H.; Guebitz G. M. Enzymatic Recovery of Polyester Building Blocks from Polymer Blends. Process Biochem. 2017, 59, 58–64. 10.1016/j.procbio.2017.01.004.

[ref28] Uekert T.; Singh A.; DesVeaux J. S.; Ghosh T.; Bhatt A.; Yadav G.; Afzal S.; Walzberg J.; Knauer K. M.; Nicholson S. R.; Beckham G. T.; Carpenter A. C. Technical, Economic, and Environmental Comparison of Closed-Loop Recycling Technologies for Common Plastics. ACS Sustain. Chem. Eng. 2023, 11 (3), 965–978. 10.1021/acssuschemeng.2c05497.

[ref29] Ronkvist Å. M.; Xie W.; Lu W.; Gross R. A. Cutinase-Catalyzed Hydrolysis of Poly(Ethylene Terephthalate). Macromolecules 2009, 42 (14), 5128–5138. 10.1021/ma9005318.

[ref30] Mueller R. J. Biological Degradation of Synthetic Polyesters-Enzymes as Potential Catalysts for Polyester Recycling. Process Biochem. 2006, 41 (10), 2124–2128. 10.1016/j.procbio.2006.05.018.

[ref31] Vertommen M. A. M. E.; Nierstrasz V. A.; Veer M. V. D.; Warmoeskerken M. M. C. G. Enzymatic Surface Modification of Poly(Ethylene Terephthalate). J. Biotechnol. 2005, 120 (4), 376–386. 10.1016/j.jbiotec.2005.06.015. 16115695

[ref32] Austin H. P.; Allen M. D.; Donohoe B. S.; Rorrer N. A.; Kearns F. L.; Silveira R. L.; Pollard B. C.; Dominick G.; Duman R.; El Omari K.; Mykhaylyk V.; Wagner A.; Michener W. E.; Amore A.; Skaf M. S.; Crowley M. F.; Thorne A. W.; Johnson C. W.; Woodcock H. L.; McGeehan J. E.; Beckham G. T. Characterization and Engineering of a Plastic-Degrading Aromatic Polyesterase. Proc. Natl. Acad. Sci. U.S.A. 2018, 115 (19), E4350–E4357. 10.1073/pnas.1718804115. 29666242 PMC5948967

[ref33] Kawai F. Emerging Strategies in Polyethylene Terephthalate Hydrolase Research for Biorecycling. ChemSusChem 2021, 14 (19), 4115–4122. 10.1002/cssc.202100740. 33949146

[ref34] Thomsen T. B.; Hunt C. J.; Meyer A. S. Standardized Method for Controlled Modification of Poly (Ethylene Terephthalate) (PET) Crystallinity for Assaying PET Degrading Enzymes. MethodsX 2022, 9, 101815 10.1016/j.mex.2022.101815. 36039192 PMC9418548

[ref35] Rezazadeh A.; Thomsen K.; Gavala H. N.; Skiadas I. V.; Fosbøl P. L. Solubility and Freezing Points of Disodium Terephthalate in Water-Ethylene Glycol Mixtures. J. Chem. Eng. Data 2021, 66 (5), 2143–2152. 10.1021/acs.jced.1c00052.

[ref36] Arnling Bååth J.; Jensen K.; Borch K.; Westh P.; Kari J. Sabatier Principle for Rationalizing Enzymatic Hydrolysis of a Synthetic Polyester. JACS Au 2022, 2 (5), 1223–1231. 10.1021/jacsau.2c00204. 35647598 PMC9131473

[ref37] Kawai F.; Kawabata T.; Oda M. Current State and Perspectives Related to the Polyethylene Terephthalate Hydrolases Available for Biorecycling. ACS Sustain. Chem. Eng. 2020, 8 (24), 8894–8908. 10.1021/acssuschemeng.0c01638.

[ref38] Siddiqui K. S.; Ertan H.; Poljak A.; Bridge W. J. Evaluating Enzymatic Productivity. The Missing Link to Enzyme Utility. Int. J. Mol. Sci. 2022, 23, 6908 10.3390/ijms23136908. 35805910 PMC9266678

[ref39] Ma S. K.; Gruber J.; Davis C.; Newman L.; Gray D.; Wang A.; Grate J.; Huisman G. W.; Sheldon R. A. A Green-by-Design Biocatalytic Process for Atorvastatin Intermediate. Green Chem. 2010, 12 (1), 81–86. 10.1039/B919115C.

[ref40] Langevin D.; Grenet J.; Saiter J. M. Moisture Sorption in Pet Influence on the Thermokinetic Parameters. Eur. Polym. J. 1994, 30 (3), 339–345. 10.1016/0014-3057(94)90297-6.

[ref41] Kawai F.; Oda M.; Tamashiro T.; Waku T.; Tanaka N.; Yamamoto M.; Mizushima H.; Miyakawa T.; Tanokura M. A Novel Ca2+-Activated, Thermostabilized Polyesterase Capable of Hydrolyzing Polyethylene Terephthalate from Saccharomonospora Viridis AHK190. Appl. Microbiol. Biotechnol. 2014, 98 (24), 10053–10064. 10.1007/s00253-014-5860-y. 24929560

[ref42] Levine H.; Slade L. Water as a Plasticizer: Physico-Chemical Aspects of Low-Moisture Polymeric Systems. Water Science Reviews 3; Cambridge University Press, 1988; pp 79–185. 10.1017/cbo9780511552083.002.

[ref43] Zhang T.; Liu S.; Li H.; Wu H.; Guo S. The Enhancement Mechanism of Flowability and Modulus of PET/TFP-Glass Composites. Polym. Compos. 2019, 40 (7), 2555–2563. 10.1002/pc.25042.

[ref44] Schubert S.; Schaller K.; Bååth J. A.; Hunt C.; Borch K.; Jensen K.; Brask J.; Westh P. Reaction Pathways for the Enzymatic Degradation of Poly(Ethylene Terephthalate): What Characterizes an Efficient PET-Hydrolase?. ChemBioChem 2023, 24 (3), e202200516 10.1002/cbic.202200516. 36399069 PMC10108200

[ref45] Clayden J.; Greeves N.; Warren S. Organic Chemistry; Oxford University Press, 2012; pp 240–267.

[ref46] Studier F. W. Protein Production by Auto-Induction in High-Density Shaking Cultures. Protein Expression Purif. 2005, 41 (1), 207–234. 10.1016/j.pep.2005.01.016. 15915565

[ref47] Zhong-Johnson E. Z. L.; Voigt C. A.; Sinskey A. J. An Absorbance Method for Analysis of Enzymatic Degradation Kinetics of Poly(Ethylene Terephthalate) Films. Sci. Rep. 2021, 11 (1), 928–929. 10.1038/s41598-020-79031-5. 33441590 PMC7806724

