# Supplementary material for: Assessment of Four Engineered PET Degrading Enzymes Considering Large-Scale Industrial Applications
Source: ACS Catal. 2023 Sep 26;13(20):13156–66. doi: 10.1021/acscatal.3c02922 (PMC10594578; doi:10.1021/acscatal.3c02922)
Supplement: Supplementary file 1 — cs3c02922_si_001.pdf [file cs3c02922_si_001.pdf]

Supporting information for

Assessment of Four Engineered PET Degrading Enzymes Considering Large-Scale Industrial Applications

Grégory Arnal[1], Julien Anglade[2], Sabine Gavalda[1], Vincent Tournier[1], Nicolas Chabot[1], Uwe T. Bornscheuer[3,], Gert Weber[4,]* and Alain Marty[1,]**

[1] Carbios, Parc Cataroux – Bâtiment B80, 8 rue de la Grolière, 63100 Clermont-Ferrand, France

[2] Toulouse Biotechnology Institute, TBI, Université de Toulouse, CNRS, INRAE, INSA, 135 avenue de Rangueil, 31077 Toulouse Cedex 4, France

[3] University of Greifswald, Institute of Biochemistry, Biotechnology & Enzyme Catalysis, Felix-Hausdorff-Str. 4, 17487 Greifswald, Germany

[4] Helmholtz-Zentrum Berlin für Materialien und Energie, Macromolecular Crystallography, Albert-Einstein-Straße 15, D-12489 Berlin, Germany

KEYWORDS

polyethylene terephthalate (PET), polyethylene terephthalate hydrolases, industrial enzymatic PET recycling, enzyme engineering, PET hydrolysis reaction conditions

* Correspondence and requests for materials should be addressed to U.T.B. (email: uwe.bornscheuer@uni-greifswald.de) or to G.W. (email: gert.weber@helmholtz-berlin.de) or to A. M. (email: Alain.Marty@carbios.com)

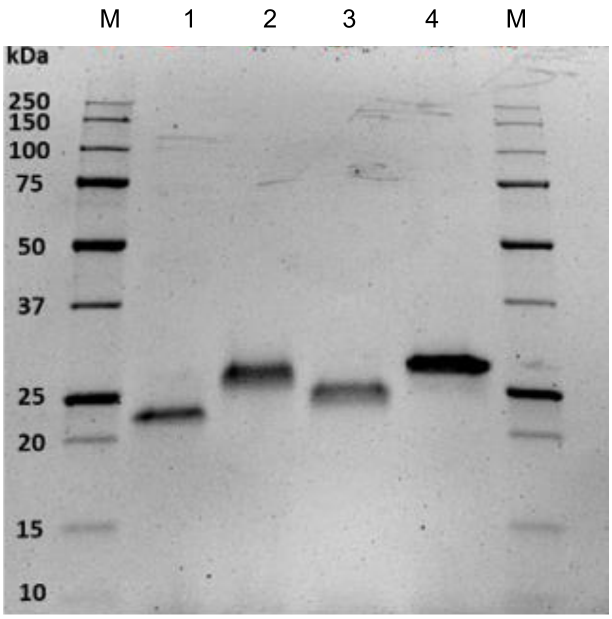

Supplementary Figure 1. SDS-PAGE analysis of 1- LCC^{ICCG}, 2- FAST-PETase, 3- HotPETase, 4- PES-H1$^{L92F/Q94Y}$. M for Protein ladder Precision Plus.

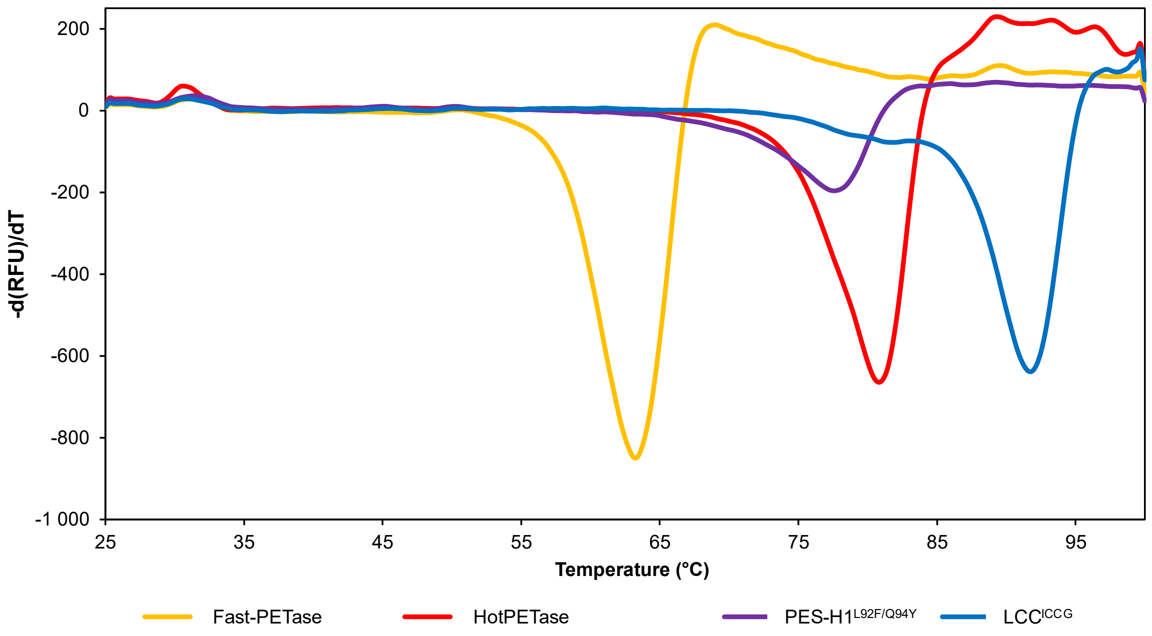

Supplementary Figure 2. DSF thermal denaturation curves for FAST-PETase (yellow), PES-H1$^{L92F/Q94Y}$ (purple) and LCC^{ICCG} (blue) in potassium phosphate buffer 100 mM, pH 8.0, and for HotPETase (red) in glycine-OH buffer 100 mM, pH 9.2.

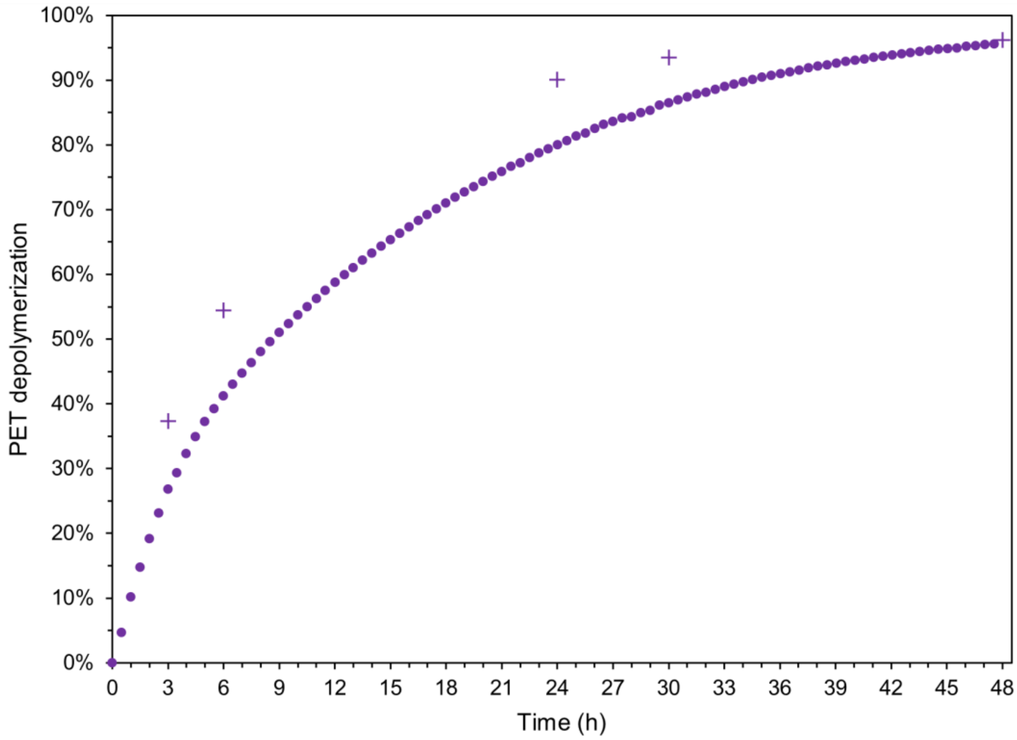

Supplementary Figure 3. 16.5% (w/w) PcW-PET depolymerization performed in a reactor using PES-H1$^{L92F/Q94Y}$ over 48 h of hydrolysis. Reaction performed at 60 °C, pH 8.0 using 16.5% (w/w) of PcW-PET and PES-H1$^{L92F/Q94Y}$ at a concentration of 1 mg_{enzyme} g_{PET}^{-1}. Purple dots represent the PET conversion in % measured by the NaOH consumption considering an exclusive production of TPA and MEG (2 mol of NaOH is consumed to titrate 1 mol of the diacid TPA). Purple crosses represent the percentage of PET conversion adjusted by considering the TPA/MHET ratio measured by UHPLC analysis at different time (1 mol of NaOH is consumed to titrate 1 mol of the monoacid MHET).

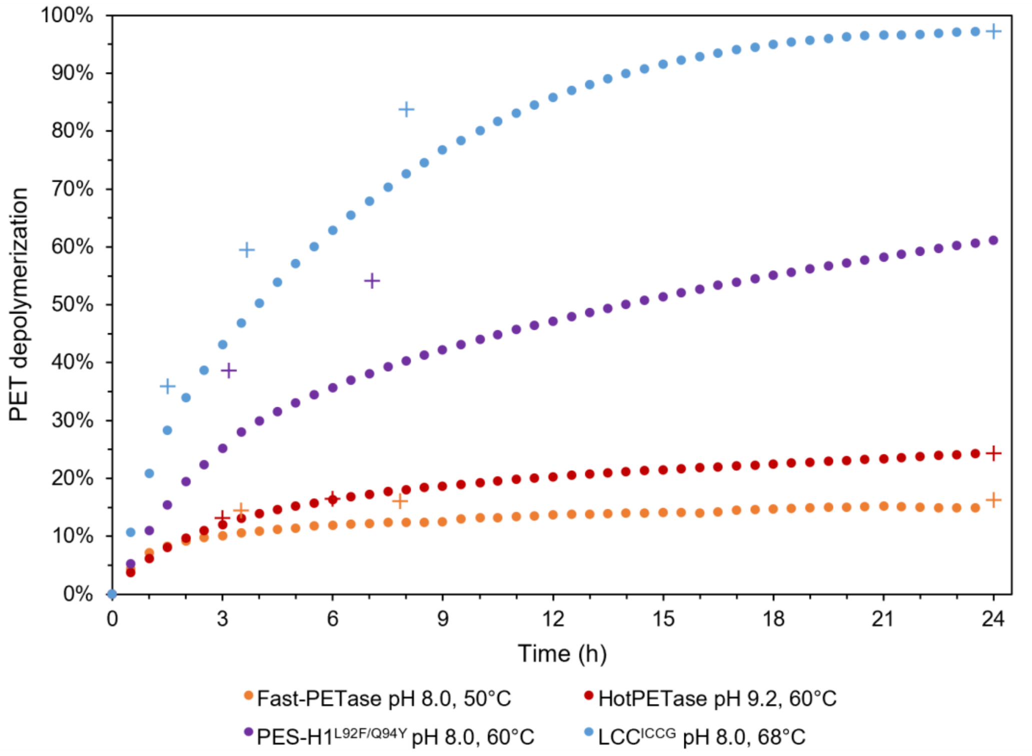

Supplementary Figure 4. Comparison of 20% (w/w) PcW-PET depolymerizations performed by the four enzymes at bioreactor scale. Enzyme-based PET depolymerizations were performed using FAST-PETase at 50 °C, pH 8.0 (orange), HotPETase at 60 °C, pH 9.2 (red), PES-H1$^{L92F/Q94Y}$ at 60 °C, pH 8.0 (purple) and LCC^{ICCG} at 68 °C, pH 8.0 (blue) of a 200 g_{PET} kg^{-1} solution with 1 mg_{enzyme} g_{PET}^{-1}. Dots represent the PET conversion in % measured by the NaOH consumption considering an exclusive production of TPA and EG (2 mol of NaOH is consumed to titrate 1 mol of the diacid TPA). Crosses represent the percentage of PET conversion adjusted by considering the TPA/MHET ratio (1 mol of NaOH is consumed to titrate 1 mol of the monoacid MHET).

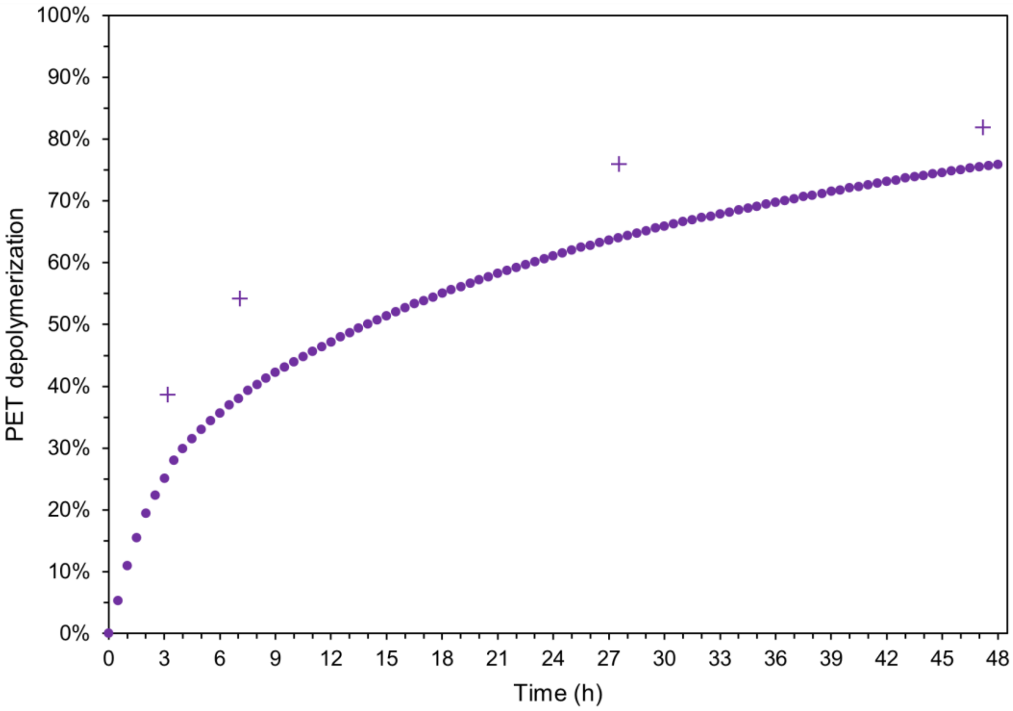

Supplementary Figure 5. 20% (w/w) PcW-PET depolymerization performed in a reactor using PES-H1$^{L92F/Q94Y}$ over 48 h of hydrolysis. Reaction performed at 60 °C, pH 8.0 using 20% (w/w) of PcW-PET and PES-H1$^{L92F/Q94Y}$ at a concentration of 1 mg_{enzyme} g_{PET}^{-1}. Purple dots represent the PET conversion in % measured by the NaOH consumption considering an exclusive production of TPA and MEG (2 mol of NaOH is consumed to titrate 1 mol of the diacid TPA). Purple crosses represent the percentage of PET conversion adjusted by considering the TPA/MHET ratio measured by UHPLC analysis at different time (1 mol of NaOH is consumed to titrate 1 mol of the monoacid MHET).

Supplementary Table 1. T_m assessments of FAST-PETase, HotPETase, PES-H1$^{L92F/Q94Y}$ and LCC^{ICCG} in 100 mM potassium phosphate buffer pH 8.0, and of HotPETase in 100 mM glycine-OH buffer pH 9.2 performed by DSF and comparison with the previously published T_m.

Enzyme	T_m assessed (°C)	T_m reported (°C)
**FAST-PETase**	63.3 ± 0.0	67.1[1] DPBS pH 7.0
**HotPETase**	80.7 ± 0.0	82.5[2] 50 mM glycine-OH, pH 9.2
**PES-H1$^{L92F/Q94Y}$**	77.6 ± 0.2	78.2[3] 50 mM potassium phosphate pH 8.0
**LCC^{ICCG}**	91.7 ± 0.2	94.0 ± 0.2[4] 20 mM tris-HCl, 300 mM NaCl, pH 8.0

Supplementary Table 2. Performances measured at different temperatures during the hydrolysis of Gf-PET for FAST-PETase, HotPETase, PES-H1$^{L92F/Q94Y}$ and LCC^{ICCG} in 100 mM potassium phosphate buffer pH 8.0, and for HotPETase in 100 mM glycine-OH buffer pH 9.2. Red hashes indicate the value of specific activity that was calculated over 2 h of reaction instead of 4 h and might be underestimated due to poor stability of the enzyme at specified temperature.

Temperature	Performance criteria	FAST-PETase	HotPETase	PES-H1$^{L92F/Q94Y}$	LCC^{ICCG}
**45 °C**	Specific activity (µmol h^{-1} mg_{enzyme}^{-1})	245.5 ± 12.5	210.9 ± 13.5	67.2 ± 0.6	55.8 ± 15.3
	PET depolymerization at 24h (%)	8.7 ± 2.3	10.4 ± 0.7	4.9 ± 0.4	5.2 ± 0.6
	PET final depolymerization (%)	9.0 ± 2.3	12.2 ± 0.9	6.6 ± 0.6	8.0 ± 0.6
**50 °C**	Specific activity (µmol h^{-1} mg_{enzyme}^{-1})	347.5 ± 46.1	429.7 ± 56.1	151.0 ± 22.4	176.7 ± 7.4
	PET depolymerization at 24h (%)	9.9 ± 3.3	15.3 ± 3.2	8.8 ± 3.1	12.8 ± 0.6
	PET final depolymerization (%)	9.6 ± 3.2	16.2 ± 3.7	11.9 ± 5.1	20.8 ± 1.7
**60 °C**	Specific activity (µmol h^{-1} mg_{enzyme}^{-1})	148.4 ± 55.6 #	1059.5 ± 35.5	369.4 ± 33.4	575.7 ± 45.8
	PET depolymerization at 24h (%)	1.5 ± 0.4	25.4 ± 3.7	10.9 ± 1.7	23.1 ± 3.4
	PET final depolymerization (%)	1.6 ± 0.4	25.0 ± 3.8	10.7 ± 1.8	28.0 ± 4.9
**65 °C**	Specific activity (µmol h^{-1} mg_{enzyme}^{-1})	20.8 ± 11.0 #	1350.8 ± 135.8 #	481.3 ± 44.7 #	752.8 ± 64.5
	PET depolymerization at 24h (%)	0.3 ± 0.4	12.7 ± 1.7	7.2 ± 1.2	31.3 ± 9.8
	PET final depolymerization (%)	0.3 ± 0.4	12.3 ± 1.5	7.0 ± 1.2	34.4 ± 10.5
**68 °C**	Specific activity (µmol h^{-1} mg_{enzyme}^{-1})	7.6 ± 3.9 #	1038.5 ± 137.7 #	404.3 ± 39.9 #	962.8 ± 28.7
	PET depolymerization at 24h (%)	0.08 ± 0.02	9.1 ± 0.8	3.9 ± 0.6	41.3 ± 5.8
	PET final depolymerization (%)	0.12 ± 0.02	8.3 ± 0.8	3.9 ± 0.6	45.8 ± 5.0

Supplementary Table 3. Performances of FAST-PETase at pH 8.0, 50 °C, HotPETase at pH 9.2, 60 °C, PES-H1$^{L92F/Q94Y}$ at pH 8.0, 60 °C and LCC^{ICCG} at pH 8.0, 68 °C during the hydrolysis of 16.5% (w/w) PcW-PET in reactors at 1 mg_{enzyme} g_{PET}^{-1}. n.a. - not applicable; n.d. - not determined.

Enzyme	Time (h)	TPA molar (%)	MHET molar (%)	Calculated PET conversion: NaOH consumption (%)	Calculated PET conversion: after TPA/MHET molar ratio adjustment (%)	Calculated PET conversion: Residual solid weight (%)
**FAST-PETase**	3.0	42	58	10.3	14.5	n.a.
	6.0	48	52	12.1	16.3	n.a.
	24.0	64	36	14.9	18.1	18.6
**HotPETase**	3.0	79	21	12.5	14.0	n.a.
	6.0	98	2	17.6	17.9	n.a.
	24.0	100	0	26.4	26.4	28.7
**PES-H1$^{L92F/Q94Y}$**	3.0	43	57	26.8	37.4	n.a.
	6.0	51	49	41.2	54.4	n.a.
	24.0	78	22	80.0	90.1	n.a.
	30.0	85	15	86.5	93.5	n.a.
	48.0	99	1	95.7	96.2	98.6
**LCC^{ICCG}**	3.0	57	43	45.3	57.6	n.a.
	6.0	71	29	67.3	78.6	n.a.
	24.0	100	0	97.3	97.3	98.1

Supplementary Table 4. Performances of FAST-PETase at pH 8.0, 50 °C, HotPETase at pH 9.2, 60 °C, PES-H1$^{L92F/Q94Y}$ at pH 8.0, 60 °C and LCC^{ICCG} at pH 8.0, 68 °C during the hydrolysis of 20% (w/w) PcW-PET in reactors at 1 mg_{enzyme} g_{PET}^{-1}. n.a. - not applicable; n.d. - not determined.

Enzyme	Time (h)	TPA molar (%)	MHET molar (%)	Calculated PET conversion: NaOH consumption (%)	Calculated PET conversion: after TPA/MHET molar ratio adjustment (%)	Calculated PET conversion: Residual solid weight (%)
FAST-PETase	3.5	45	55	10.5	14.5	n.a.
	7.8	54	46	12.4	16.1	n.a.
	24.0	83	17	14.9	16.3	16.3
HotPETase	3.0	82	18	12.0	13.2	n.a.
	6.0	98	2	16.3	16.5	n.a.
	24.0	100	0	24.3	24.3	24.1
PES-H1$^{L92F/Q94Y}$	3.2	35	64	25.8	38.6	n.a.
	7.1	43	56	38.4	54.2	n.a.
	24.0	n.d.	n.d.	61.1	n.d.	n.a.
	27.5	68	32	64.0	75.9	n.a.
	47.2	85	14	75.6	81.9	81.1
LCC^{ICCG}	1.5	57	43	28.3	35.9	n.a.
	3.7	61	39	47.8	59.5	n.a.
	8.0	73	27	72.5	83.7	n.a.
	24.0	100	0	97.2	97.2	98.1

Supplementary Table 5. Productivities of the four different PET hydrolases using 20% (w/w) post-consumer colored-flake PET waste powder (PcW-PET) as substrate.

Enzyme	Maximum productivity [g_{TPAeq} L^{-1} h^{-1}]	Average productivity [g_{TPAeq} L^{-1} h^{-1}]
**FAST-PETase**	15.9	1.1[a]
**HotPETase**	17.0	1.8[a]
**PES-H1$^{L92F/Q94Y}$**	17.3	4.4[a]; 2.7[b]
**LCC^{ICCG}**	34.5	7.0[a]

[a] at 24 h. [b] at 48 h.

Nucleotide and expressed amino sequences for production of LCC^{ICCG}, FAST-PETase, HotPETase and PES-H1$^{L92F/Q94Y}$. All nucleotide sequences were codon-optimized for expression in *E. coli* and were inserted between *NdeI* and *XhoI* restriction enzyme sites of pET-26b(+) bacterial expression plasmid. Codons mutated from the wild type enzyme are indicated in red.

LCC^{ICCG} nucleotide sequence:

ATGAGCAACCCGTACCAGCGTGGCCCGAATCCGACCCGCAGCGCACTGACCGCAGATGGCCCGTTTAGCGTGGCA

ACCTACACCGTCTCACGCCTGTCAGTCTCGGGTTTTGGCGGTGGCGTGATTTATTACCCGACCGGCACGTCTCTG

ACGTTCGGTGGCATCGCGATGAGTCCGGGTTATACCGCAGATGCTAGCTCTCTGGCATGGCTGGGTCGTCGCCTG

GCTTCCCATGGCTTTGTGGTTCTGGTGATTAACACGAATTCACGTTTCGATGGCCCGGACAGCCGCGCCTCTCAG

CTGAGTGCCGCCCTGAACTACCTGCGTACCAGTTCCCCGAGCGCCGTTCGCGCACGTCTGGATGCAAATCGTCTG

GCGGTTGCCGGTCATTCTATGGGTGGCGGTGGCACCCTGCGTATTGCAGAACAAAACCCGAGCCTGAAAGCGGCT

GTCCCGCTGACCCCGTGGCACACCGATAAAACGTTTAATACCAGTGTCCCGGTGCTGATTGTTGGCGCAGAAGCT

GACACCGTGGCGCCGGTTTCGCAGCATGCCATCCCGTTTTATCAAAACCTGCCGAGCACCACGCCGAAAGTTTAC

GTCGAACTGTGCAACGCATCGCACATTGCTCCGAATAGCAACAATGCGGCCATTTCCGTTTATACGATCTCATGG

ATGAAACTGTGGGTCGATAATGACACCCGTTACCGCCAGTTCCTGTGTAATGTGAACGACCCGGCTCTGTGCGAC

TTCCGCACCAATAATCGCCACTGCCAACTCGAGCACCACCACCACCACCACTGA

LCC^{ICCG} expressed amino acid sequence (amino acid numbering starts at 35):

M^{35}SNPYQRGPNPTRSALTADGPFSVATYTVSRLSVSGFGGGVIYYPTGTSLTFGGIAMSPGYTADASSLAWLGRR

LASHGFVVLVINTNSRFDGPDSRASQLSAALNYLRTSSPSAVRARLDANRLAVAGHSMGGGGTLRIAEQNPSLKA

AVPLTPWHTDKTFNTSVPVLIVGAEADTVAPVSQHAIPFYQNLPSTTPKVYVELCNASHIAPNSNNAAISVYTIS

WMKLWVDNDTRYRQFLCNVNDPALCDFRTNNRHCQLEHHHHHH

FAST-PETase nucleotide sequence with a pelB leader sequence (lowercase) upfront the nucleotide sequence encoding for the mature protein (uppercase):

atgaaatacctgctgccgaccgctgctgctggtctgctgctcctcgctgcccagccggcgatggccCAGACCAAT

CCATACGCTCGTGGTCCAAATCCGACCGCCGCAAGCCTGGAAGCAAGCGCAGGTCCATTTACCGTTCGCAGCTTT

ACCGTTAGCCGTCCAAGCGGTTATGGTGCAGGTACCGTTTATTATCCGACCAATGCAGGTGGCACCGTTGGTGCA

ATTGCTATTGTTCCGGGTTATACCGCCCGCCAGAGCAGCATTAAATGGTGGGGTCCGCGCCTGGCCAGTCATGGT

TTTGTTGTTATTACCATTGATACCAATAGCACCCTGGATCAGCCGGAAAGCCGTTCAAGTCAGCAGATGGCAGCA

CTGCGTCAGGTGGCGTCTCTGAATGGTACTAGTAGCAGTCCGATTTATGGTAAAGTTGATACCGCACGTATGGGC

GTTATGGGTTGGAGTATGGGTGGTGGTGGTAGTCTGATTAGTGCCGCTAATAATCCGAGCCTGAAAGCAGCGGCA

CCGCAGGCACCGTGGCATAGCAGTACCAACTTTAGTAGCGTTACGGTTCCGACCCTGATTTTTGCTTGTGAAAAT

GATAGCATTGCACCGGTTAATAGCAGCGCACTGCCGATTTATGATTCAATGAGCCAGAATGCAAAACAGTTTCTG

GAAATTAAAGGCGGTAGCCATTCTTGTGCCAATAGTGGTAATAGCAATCAGGCACTGATTGGTAAAAAGGGTGTT

GCCTGGATGAAACGTTTTATGGATAACGATACCCGTTATAGCACCTTTGCATGTGAAAATCCGAATAGTACCGCC

GTTAGTGATTTTCGCACCGCAAATTGCAGTCTCGAGCACCACCACCACCACCACTGA

FAST-PETase expressed amino acid sequence (amino acid numbering starts at 28):

Q^{28}TNPYARGPNPTAASLEASAGPFTVRSFTVSRPSGYGAGTVYYPTNAGGTVGAIAIVPGYTARQSSIKWWGPRL

ASHGFVVITIDTNSTLDQPESRSSQQMAALRQVASLNGTSSSPIYGKVDTARMGVMGWSMGGGGSLISAANNPSL

KAAAPQAPWHSSTNFSSVTVPTLIFACENDSIAPVNSSALPIYDSMSQNAKQFLEIKGGSHSCANSGNSNQALIG

KKGVAWMKRFMDNDTRYSTFACENPNSTAVSDFRTANCSLEHHHHHH

HotPETase nucleotide sequence:

ATGCAGACTAACCCCTATGCTCGCGGGCCGAATCCTACAGCGGCCTCGTTGGAAGCCAGTGCAGGTCCCTTCACC

GTACGTTCGTTTACTGTTGCGCGTCCAGTGGGATATGGGGCTGGCACCGTCTATTATCCGACTAACGCCGGTGGT

ACTGTGGGCGCAATCGCCATTGTCCCCGGCTACACTGCAACTCAATCCTCGATTAATTGGTGGGGACCACGCTTG

GCTAGCCACGGGTTTGTTGTGATCACCATCGATACTAACAGTACGTTGGACAAGCCAGAGAGTCGCAGCTCTCAG

CAGATGGCGGCATTACGCCAGGTGGCGAGCTTAAATGGGACGAGTTCAAGTCCAATTTATGGCAAGGTCGATACC

GCCCGTGGTGGAGTAATGGGCTGGAGTATGGGTGGCGGCGGATCTCTTATCTCGGCAGCGAATAATCCAAGCCTG

AAGGCAGCAGCCGTTATGGCACCCTGGCATAGTAGTACGAACTTTTCGTCTGTTACGGTTCCTACGCTTATCTTT

GCCTGTGAGAATGATAGGATCGCACCAGTGAAGGAGTACGCCTTGCCAATTTACGACTCGATGTCGCTCAACGCT

AAACAGTTTCTTGAGATTTGTGGGGGCTCTCACTCTTGTGCCTGTAGTGGGAATTCCAATCAAGCCCTTATCGGA

ATGAAGGGCGTTGCTTGGATGAAGCGCTTCATGGACAATGACACTCGTTATTCACAGTTCGCTTGCGAAAACCCA

AACTCAACCGCCGTATGTGATTTTCGCACTGCTAACTGCAGCCTCGAGCACCACCACCACCACCACTGA

HotPETase expressed amino acid sequence (amino acid numbering starts at 27):

M^{27}QTNPYARGPNPTAASLEASAGPFTVRSFTVARPVGYGAGTVYYPTNAGGTVGAIAIVPGYTATQSSINWWGPR

LASHGFVVITIDTNSTLDKPESRSSQQMAALRQVASLNGTSSSPIYGKVDTARGGVMGWSMGGGGSLISAANNPS

LKAAAVMAPWHSSTNFSSVTVPTLIFACENDRIAPVKEYALPIYDSMSLNAKQFLEICGGSHSCACSGNSNQALI

GMKGVAWMKRFMDNDTRYSQFACENPNSTAVCDFRTANCSLEHHHHHH

PES-H1$^{L92F/Q94Y}$ nucleotide sequence:

ATGGCGAACCCGTACGAGCGCGGGCCCGATCCCACCGAGTCGAGCATCGAGGCCGTCCGCGGGCCGTTCGCCGTG

GCCCAGACGACGGTGTCGAGGCTCCAGGCCGACGGCTTCGGCGGCGGGACCATCTACTACCCGACCGACACGAGC

CAGGGCACCTTCGGTGCGGTGGCGATCTCGCCGGGGTTCACGGCGGGCCAGGAGAGCATCGCCTGGCTCGGCCCC

CGCATCGCGTCGCAGGGCTTCGTGGTGATCACGATCGACACGATCACGCGCTTCGACTATCCCGACAGCCGGGGT

CGCCAGCTGCAGGCCGCGCTCGACCACCTGCGCACCAACAGCGTCGTGCGCAACCGGATCGACCCGAACCGGATG

GCGGTCATGGGCCACTCGATGGGCGGCGGCGGGGCGCTGTCCGCCGCGGCGAACAACACGAGCCTCGAGGCCGCC

ATCCCGCTGCAGGGCTGGCACACCCGGAAGAACTGGTCGAGCGTGCGGACGCCGACCCTGGTGGTCGGGGCCCAG

CTCGACACCATCGCGCCGGTGAGCTCGCACTCGGAGGCCTTCTACAACAGCCTGCCGAGCGACCTCGACAAGGCG

TACATGGAGCTCCGCGGGGCCAGCCACCTCGTGTCGAACACGCCCGACACGACGACCGCGAAGTACAGCATCGCC

TGGCTCAAGCGGTTCGTCGACGACGACCTCCGCTACGAGCAGTTCCTGTGCCCGGCGCCGGACGACTTCGCGATC

TCCGAGTACCGCTCCACCTGCCCGTTCCTCGAGCACCACCACCACCACCACTGA

PES-H1$^{L92F/Q94Y}$ expressed amino acid sequence (amino acid numbering starts at 0):

M^{0}ANPYERGPDPTESSIEAVRGPFAVAQTTVSRLQADGFGGGTIYYPTDTSQGTFGAVAISPGFTAGQESIAWLG

PRIASQGFVVITIDTITRFDYPDSRGRQLQAALDHLRTNSVVRNRIDPNRMAVMGHSMGGGGALSAAANNTSLEA

AIPLQGWHTRKNWSSVRTPTLVVGAQLDTIAPVSSHSEAFYNSLPSDLDKAYMELRGASHLVSNTPDTTTAKYSI

AWLKRFVDDDLRYEQFLCPAPDDFAISEYRSTCPFLEHHHHHH

Bibliography

(1) Lu, H.; Diaz, D. J.; Czarnecki, N. J.; Zhu, C.; Kim, W.; Shroff, R.; Acosta, D. J.; Alexander, B. R.; Cole, H. O.; Zhang, Y.; Lynd, N. A.; Ellington, A. D.; Alper, H. S. Machine Learning-Aided Engineering of Hydrolases for PET Depolymerization. *Nature* **2022**, *604* (7907), 662–667. https://doi.org/10.1038/s41586-022-04599-z.

(2) Bell, E. L.; Smithson, R.; Kilbride, S.; Foster, J.; Hardy, F. J.; Ramachandran, S.; Tedstone, A. A.; Haigh, S. J.; Garforth, A. A.; Day, P. J. R.; Levy, C.; Shaver, M. P.; Green, A. P. Directed Evolution of an Efficient and Thermostable PET Depolymerase. *Nat. Catal. 2022 58* **2022**, *5* (8), 673–681. https://doi.org/10.1038/s41929-022-00821-3.

(3) Pfaff, L.; Gao, J.; Li, Z.; Jäckering, A.; Weber, G.; Mican, J.; Chen, Y.; Dong, W.; Han, X.; Feiler, C. G.; Ao, Y. F.; Badenhorst, C. P. S.; Bednar, D.; Palm, G. J.; Lammers, M.; Damborsky, J.; Strodel, B.; Liu, W.; Bornscheuer, U. T.; Wei, R. Multiple Substrate Binding Mode-Guided Engineering of a Thermophilic PET Hydrolase. *ACS Catal.* **2022**, *12* (15), 9790–9800. https://doi.org/10.1021/acscatal.2C02275.

(4) Tournier, V.; Topham, C. M.; Gilles, A.; David, B.; Folgoas, C.; Moya-Leclair, E.; Kamionka, E.; Desrousseaux, M. L.; Texier, H.; Gavalda, S.; Cot, M.; Guémard, E.; Dalibey, M.; Nomme, J.; Cioci, G.; Barbe, S.; Chateau, M.; André, I.; Duquesne, S.; Marty, A. An Engineered PET Depolymerase to Break down and Recycle Plastic Bottles. *Nature* **2020**, *580* (7802), 216–219. https://doi.org/10.1038/s41586-020-2149-4.
